# Outcomes of COVID-19 Patients under Cytotoxic Cancer Chemotherapy in Brazil

**DOI:** 10.3390/cancers12123490

**Published:** 2020-11-24

**Authors:** Mateus Bringel Oliveira Duarte, Frederico Leal, Juliana Luz Passos Argenton, José Barreto Campello Carvalheira

**Affiliations:** 1 Division of Oncology, Department of Internal Medicine, Faculty of Medical Sciences, Universidade Estadual de Campinas (UNICAMP), Campinas 13083-887, Brazil; m190430@dac.unicamp.br (M.B.O.D.); fleal@unicamp.br (F.L.); 2 Fundação de Desenvolvimento da Universidade Estadual de Campinas (FUNCAMP), Campinas 13083-851, Brazil; jpassos@unicamp.br

**Keywords:** neoplasms, COVID-19, drug therapy, comorbidity, propensity score

## Abstract

**Simple Summary:**

Cancer patients present a distinct vulnerability to COVID-19. It is unclear if chemotherapy per se increases the overall risk in this population. In our study, we analyzed retrospective COVID-19 data linked to oncological information systems to evaluate outcomes in COVID-19 cancer patients under chemotherapy. We identified 681 patients with a past history of chemotherapy. Patients in active chemotherapy did not have an increased mortality compared to non-active chemotherapy COVID-19 cases. We identified the use of topoisomerase II inhibitors and alkylating agents as protective factors, while palliative intent of treatment and hematological neoplasms were risk factors.

**Abstract:**

Background: Cancer patients present a distinct vulnerability to COVID-19. It is unclear if chemotherapy could accentuate the overall risk in these patients. Methods: We performed a retrospective analysis linking COVID-19 data and oncological information systems to compare lethality in patients undergoing cytotoxic chemotherapy before COVID-19. We considered patients who received chemotherapy in the last 30 days as in “active treatment”, and patients who did not receive drugs in this period as “non-active treatment” for propensity-score pair matching. We also tested the influence of baseline variables in our results in a multivariate model. Results: 66.1% (162/246) of patients in matched active chemotherapy died vs. 70.2% (172/246) in the matched non-active chemotherapy group. The risk of death was positively associated with palliative intent of treatment and hematologic neoplasms. Being in active chemotherapy was not associated with increased mortality compared to non-active treatment. We also noted in exploratory propensity-score matchings that the use of alkylating agents (odds ratio [OR] 0.38, 95% confidence interval [CI], 0.21–0.70) and topoisomerase II inhibitors (OR 0.28, 95% CI 0.14–0.56) were protective factors. Conclusions: This study does not demonstrate an increase in mortality for cancer patients under active cytotoxic chemotherapy with COVID-19.

## 1. Introduction

The new SARS-CoV-2 infection (COVID-19) has already caused more than 800,000 deaths worldwide, with 100,000 in Brazil alone [1]. Age, cardiovascular disease, chronic lung diseases, and diabetes have been described as the main risk factors that determine the severity of COVID-19 [2,3]. In parallel, there is a growing body of evidence that cancer also negatively impacts outcomes in patients with COVID-19 [4,5,6]. Moreover, the pandemic has already significantly influenced patient flow in cancer centers, with some patients and physicians opting for deferral of the treatment due to the underlying increased risk and susceptibility to COVID-19 [5,7]. Therefore, establishing the role of anti-cancer treatments in COVID-19 outcomes is an urgent unmet need.

Cancer patients face a unique condition during COVID-19 [8,9]. Risk factors associated with cancer itself may be predictors of COVID-19 severity. At the same time, factors related to cancer treatment may also impact outcomes for patients who develop COVID-19. Early studies by Tian et al. [10] and Yang et al. [11] demonstrated that chemotherapy use in the last 4 weeks negatively impacts the outcomes of COVID-19. In accordance, Lee et al. [12] reported for the UK Coronavirus Cancer Monitoring Project (UKCC) that chemotherapy within 4 weeks of COVID-19 infection was associated with an unfavorable prognosis in hematologic patients. On the other hand, recent cytotoxic chemotherapy was not associated with worse prognosis from COVID-19 in three large studies [13,14,15]. Altogether, these studies indicate that the heterogeneity of both the study population and chemotherapy type influenced the divergent effects of chemotherapy on COVID-19 prognosis.

Since patients under chemotherapy represent a small fraction of COVID-19 patients, analysis of large samples of this population subgroup are still lacking granularity [12,13,14,16]. Herein, we aimed to explore the prognostic impact of cytotoxic chemotherapy in patients with COVID-19 in a retrospective Brazilian registry cohort. We also attempted to examine the influence of individual chemotherapeutic drug classes in the outcomes of these patients with COVID-19.

## 2. Results

### 2.1. Participants

**Figure 1:**
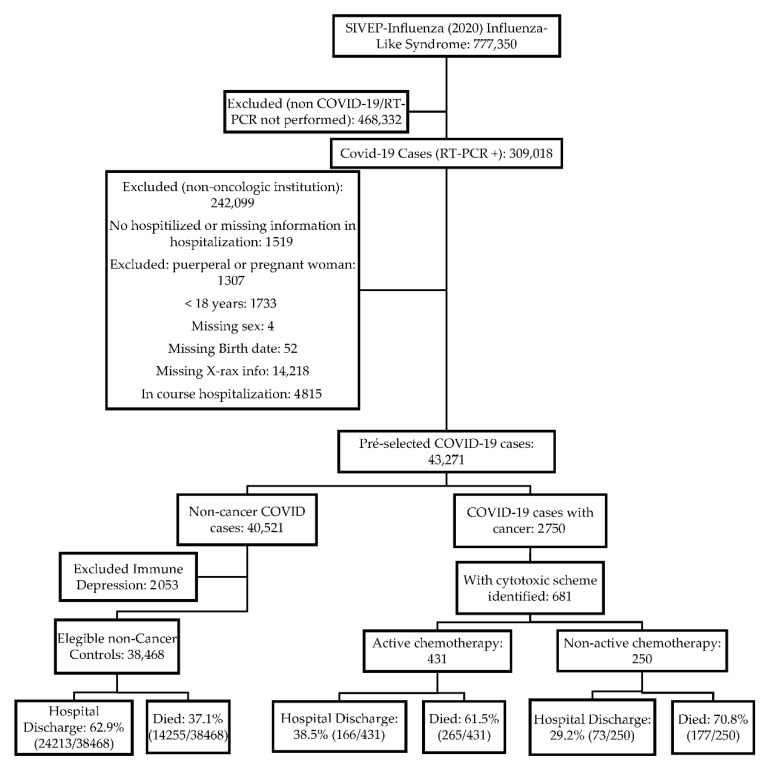
Data flowchart for the analysis process.

As of 28 September 2020, there were 777,350 flu-like syndromes registered in the Sistema Informatizado de Vigilância Epidemiológica (SIVEP) databank. We selected 309,018 patients who had a positive RT-PCR test for SARS-CoV-2. Subsequently, we excluded a total of 265,747 cases, 242,099 due to the reporting in non-oncologic or nonacademic institutions, 1519 due to outpatient treatment or missing information in hospitalization, 1307 to woman in pregnant or puerperal periods, 1733 to patients of ages younger than 18 years, 4 to missing sex info, 14,218 to missing X-rays (criterion for good quality data) and birthdate (used in the linkage process), and 4815 due to in-course hospitalization (without a defined outcome). Among the remaining 43,271 patients, we identified 2750 with cancer, 2053 with immune depression, and 38,468 eligible non-cancer controls. After the linkage process, we identified 991 cases of COVID-19 in the Sistema de Informação Ambulatorial (SIA—outpatient information system) databank, in which 681 had entries for cytotoxic chemotherapy. After the propensity score pair matching process between non-cancer eligible controls and patients under cytotoxic chemotherapy (active and non-active), we obtained 681 matched controls (Table S1, Figure S1). Of the patients, 63.3% (431/681) were in active chemotherapy, and 36.7% (250/681) were in a non-active treatment group. Further, 61.5% (265/681) of patients under active chemotherapy and 70.8% (177/250) in non-active chemotherapy died, while 37.9% (217/681) of non-cancer matched controls presented this outcome. Figure 1 represents the data flowchart.

### 2.2. Descriptive Data

The majority of the cohort were individuals from the Brazilian Southeast Region, representing 51.9% (19,955/38,468) of non-cancer eligible controls, 65.2% (281/431) of active chemotherapy, and 55.2% of non-active chemotherapy group. Only 26.5% (10,192/38,468) of eligible non-cancer controls, 28.1% (121/431) of active chemotherapy, and 18.4% (46/250) of the non-active chemotherapy group were less than 50 years old. Cardiovascular disease was the most reported comorbidity in both eligible non-cancer controls (41.2%, 15,866/38,468), active chemotherapy (19.3%, 83/431), and non-active chemotherapy cases (24.0%, 60/250). Critical forms of COVID-19 infection presented in 25.6% (9848/38,468) of the eligible non-cancer controls, 22.5% (97/431) of active chemotherapy, and 24.4% (61/250) of non-active chemotherapy cases. After matching, we observed an important reduction in the imbalance between active chemotherapy cases and non-active chemotherapy controls, and there was no significant difference between cases and pair-matched controls (Table 1, Figures S1–S3). Comorbidities distribution according to cancer type is shown in Tables S2 and S3.

Among patients under cytotoxic chemotherapy, we noted that most presented stage IV disease (63.1%, 272/431, active chemotherapy; 68.8%, 172/250, non-active chemotherapy), received doublet or triplet regimens (55.0%, 237/431, active chemotherapy; 52.8%, 132/250, non-active chemotherapy), and were in first-line palliative treatment (44.8%, 193/431, active chemotherapy; 42.0%, 105/250, non-active chemotherapy). Gastrointestinal (28.1%, 121/431, active chemotherapy; 31.2%, 78/250, non-active chemotherapy) and hematologic (25.7%, 111/431, active chemotherapy; 17.6%, 44/250, non-active chemotherapy) were the most common primary sites. Cisplatin was the most frequently used drug (43.2%, 186/431, active chemotherapy; 50.8%, 127/250, non-active chemotherapy), followed by anti-metabolite (39.2%, 169/431, active chemotherapy; 38.4%, 96/250, non-active chemotherapy), microtubule inhibitor (35.5%, 153/431, active chemotherapy; 34.4%, 86/250, non-active chemotherapy), and alkylating drugs (26.7%, 153/431, active chemotherapy; 21.2%, 53/250, non-active chemotherapy). These findings are represented in Table 1.

### 2.3. Outcome Data

**Figure 2:**
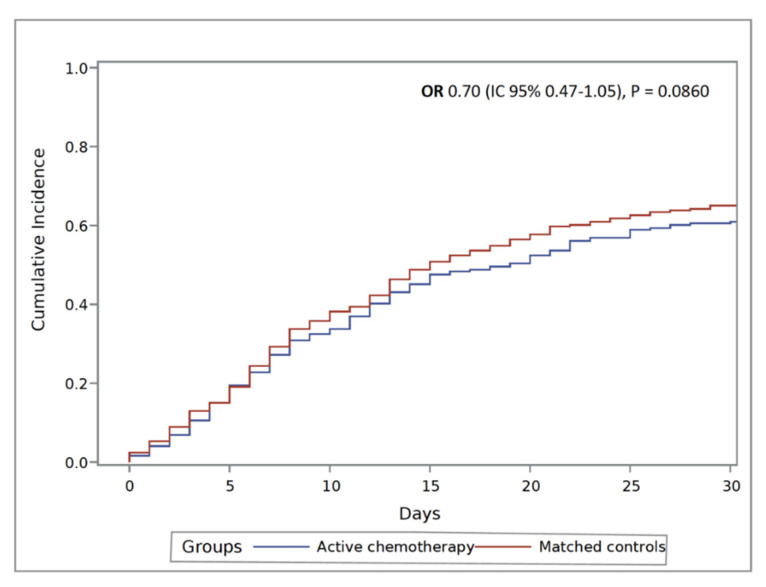
Cumulative incidence of death in cancer patients under active chemotherapy and non-active chemotherapy pair-matched controls.

After propensity score matching between active and non-active chemotherapy groups, 66.1% (162/248) of patients in the active chemotherapy group died compared to 70.2% (172/248) in the non-active chemotherapy group. As shown in Figure 2, this difference was not statistically significant (OR 1.18, 95% CI 0.95–1.46, *p* = 0.1306).

In an univariate analysis for risk of death for patients undergoing cytotoxic chemotherapy, we observed that use of alkylating agents (odds ratio [OR] 0.62, 95% confidence interval [CI] 0.44–0.89, *p* = 0.0092), use of cisplatin (OR 1.40, 95% CI 1.02–1.92, *p* = 0.0388), use of topoisomerase 2 inhibitor (OR 0.49, 95% CI 0.38–0.73, *p* = 0.0003), line of treatment (palliative first line, OR 1.90, 95% CI 1.28–2.81, *p* = 0.0013; palliative second line, OR 2.31, 95% CI 1.20–4.49, *p* = 0.0127), and active chemotherapy (OR 0.66, 95% CI 0.47–0.92, *p* = 0.0144) were associated with survival outcomes (Table 2). In multivariate analysis, use of topoisomerase 2 inhibitor (OR 0.55, 95% CI 0.36–0.85, *p* = 0.0070), line of treatment (palliative first line, OR 1.83, 95% CI 1.21–2.74, *p* = 0.0038; palliative second line, OR 2.62, 95% CI 1.15–4.45, *p* = 0.0180); hematological (OR 1.68, 95% CI 1.05–2.95, *p* = 0.0310), and active chemotherapy (OR 0.66, 95% CI 0.46–0.93, *p* = 0.0169) remained associated with death after the backward elimination process. We found no significant interaction between the use of topoisomerase 2 inhibitor and the active use of chemotherapy in our multivariate final model.

**Figure 3:**
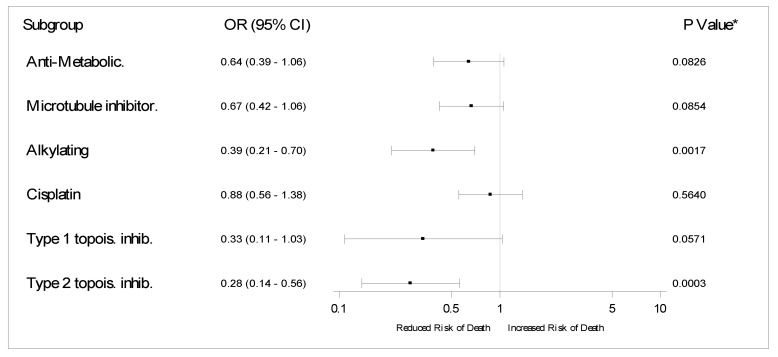
Summary of propensity score pair matching by drug classes. Each row represents a unique propensity score pair matching. The variable balances can be accessed by drug in Supplementary Tables. Abbreviations: Inhib: Inhibitor; Topois: Topoisomerase. * Wald conditional logistic regression *p*-value.

To further investigate the effect of drugs classes, we ran a second propensity score matching to compare patients under active chemotherapy receiving anti-metabolic, microtubule inhibitor, alkylating, cisplatin, topoisomerase 1 inhibitors, and topoisomerase 2 against their respective non-active chemotherapy controls (Figures S4–S9, Tables S4–S9). We found that use of alkylating agents (OR 0.38, 95% CI 0.21–0.70, *p* = 0.0017) and use of topoisomerase 2 inhibitors (OR 0.28 95% CI 0.14–0.56, *p* = 0.0003) were protective factors. Figure 3 summarizes these findings.

## 3. Discussion

To our knowledge, this is one of the largest cohorts of patients with COVID-19 and under chemotherapy studied so far. In our analysis, patients receiving antineoplastic chemotherapy were at increased risk of death from COVID-19. This effect persisted after adjusting for confounding factors with propensity score matching. In addition, patients under active treatment (those who had received cytotoxic chemotherapy in the last 30 days) were at similar risk of death when compared to those whose treatment was currently inactive. It is difficult to determine if this result arises from a real benefit from chemotherapy or from selection bias due to deferring treatment delivery for patients with low survival expectancy and/or poor performance. It is worth noting that, before matching, our cancer cohort presented with an older age, predominantly female sex, and a lower number of comorbidities. Non-oncological variables such as Brazilian region, and age itself, were also associated with survival outcomes.

Data linkage between a COVID-19 registry and a cancer treatment database provides a powerful weapon to understand real-world COVID-19 populations. Saliently, our inclusion criteria may have resulted in selection biases, for patients under chemotherapy may not adequately represent the overall cancer patient population. In the COVID-19 and cancer consortium (CCC19) cohort [14], a worldwide study of COVID-19 patients with a current or past cancer diagnosis, the authors reported a proportion of 22% of hematologic malignancies, which is close to the 22.8% reported in our study. However, the authors reported a lower proportion of gastrointestinal malignancies (12%), against 29.2% in our data. A possible explanation for this discrepancy is the longer period of chemotherapy that some gastrointestinal tumors are often submitted to, resulting in a greater likelihood of these being selected by our data linkage process. Due to the exclusion of hormone therapy from our study, hormone-sensitive neoplasms were also underrepresented, with prostate cancer representing only 2.5% of the population analyzed.

Even though cancer patients in our cohort presented a high mortality rate (64.9%), relatively few were submitted to invasive mechanical ventilation (23.2%), with no statistical difference between active and non-active chemotherapy before the matching process. Previous studies have reported that cancer patients are less often submitted to invasive protocols and intensive care admissions than non-cancer patients [17]. In our study, only a third of patients who died were submitted to invasive mechanical ventilation, against 53% of the non-cancer controls before matching. We cannot rule out that the shortage of ICU beds in the COVID-19 Brazil pandemic could lead to limited access to invasive procedures for patients with cancer.

Since the beginning of the pandemic there have been concerns that chemotherapy could impact on COVID-19 lethality. Early results of studies from China [10,11] pointed to an increased risk in this subgroup of patients. More recent analyses from the US [13] and worldwide [14], however, did not show worse outcomes for patients receiving chemotherapy [18,19]. In the UK study [20], the authors reported that patients submitted to non-palliative regimens (neoadjuvant, adjuvant, and radical) were associated with better outcomes compared to palliative lines. Consistently, after adjusting for confounding factors, we also observed an association between the line of chemotherapy and survival outcomes. The death rate difference between these studies is noteworthy: While the UK cohort [20] found a lethality rate in hospitalized COVID-19 patients undergoing chemotherapy of 27%, Yang et al. [11] and Jee et al. [13] found 48% and 21%, respectively, and our study found 64.9%. Hospitalized COVID-19 patients in Brazil in general have poor outcomes, suggesting that COVID-19 patients are hospitalized with more severe presentations in Brazil. Consistent with this idea, Baqui et al. [21] reported 47% lethality in non-cancer patients in a previous SIVEP study. The same study also reported a high rate of deaths outside an ICU. In our study, controls before matching presented 37.1% lethality.

An Italian retrospective cohort [22], with 582 patients, reported that patients with acute myeloid leukemia, lymphomas, and plasma cell neoplasms were at an increased risk of death, particularly for those with progressive disease. These findings were consistent with two prospective cohorts [12,23], in which leukemia was the only primary tumor associated with an increased risk of death from COVID-19. In accordance, we also found a worst prognosis for patients with hematological cancers compared to other neoplasms.

The associations between topoisomerase 2 inhibitor and alkylating agents use and better outcomes are provocative. To analyze the individual effect of drugs in patients with COVID-19, a higher level of granularity is required. In our exploratory analyses, 134 and 168 patients used topoisomerase 2 inhibitors and alkylating agents before COVID-19, 94 and 115 of whom were in active chemotherapy during infection, respectively. After the propensity score pair matching, those undergoing active treatment appeared to benefit from this effect. Similarly, Suárez et al. [23] showed a protective effect of alkylating drugs in a prospective hematologic cohort with COVID-19. No other analyzed drug was associated with survival in our analysis. It is difficult to state if this finding is due to a drug anti-viral effect or to a selection bias. Recent studies have identified a possible activity of doxorubicin against SARS-CoV-2 [24,25,26]. A potential therapeutic effect of cyclophosphamide for respiratory failure caused by COVID-19 has been hypothesized [27], but in animal models this drug has been used to exacerbate COVID-19 infection [28]. We hope that further studies may clarify these findings.

Delivering cytotoxic chemotherapy during a pandemic has been challenging [29]. Both oncologists and patients may be concerned that chemotherapy-induced immunosuppression may increase risks of unfavorable outcomes from COVID-19 [30]. Going to a cancer center to receive treatment, and therefore increasing the likelihood of being exposed to infection, also concerns many patients and doctors. While our cohort showed that cancer patients are indeed at an increased risk of death from COVID-19, our data does not suggest active chemotherapy as an independent risk factor. Our results suggest cancer patients should not be denied cytotoxic chemotherapy on the sole ground of fear of COVID-19. Differential effects of different primary tumors and cytotoxic drugs on COVID-19 outcomes should be further investigated.

## 4. Materials and Methods

### 4.1. Study Design

We designed a retrospective cohort study with a propensity score pair-matched analysis to evaluate the outcomes of patients with COVID-19 under cytotoxic chemotherapy for cancer treatment. Initially, in order to identify patients under chemotherapy presenting with COVID-19 infection, we performed a linkage between two databases of the Brazilian public health system (SUS).

Information about COVID-19 and baseline patient information was extracted from SIVEP, which is the Brazilian Ministry of Health’s electronic registry for epidemiological surveillance of flu-like syndromes. The Brazilian Ministry of Health adapted SIVEP, originally developed for influenza vigilance, to account for epidemiological and clinical data of COVID-19 patients in Brazil. Following Brazilian national guidelines for epidemiological surveillance of COVID-19, all patients with acute respiratory syndromes admitted to any Brazilian hospital must be reported. The Brazilian Health Ministry determines that all Brazilian states investigate and register these patients in SIVEP daily [31]. The SIVEP registry contains not only data about the incidence and number of cases of acute respiratory distress syndromes but also clinical presentation, inpatient admission, intensive care unit admission, comorbidities, use of mechanical ventilation (invasive and non-invasive), and clinical outcomes. It is worth noting that identification fields in the SIVEP case report form are mandatory, while those related to clinical characteristics are not. The SIVEP databank is dynamic and is generated and managed by local teams during the hospitalization of all patients with acute respiratory distress syndrome. Dedicated Brazilian Health Surveillance Secretariat teams clean and anonymize SIVEP data before making it available online. All information is tested for integrity, internal validation, and exclusion of duplicates. SIVEP has mainly been employed for Brazilian anti-COVID-19 strategical planning.

Information about oncologic variables was extracted from SIA. In SUS, the public funding for outpatient treatments (including chemotherapy to cancer treatment) is performed by the SIA. These datasets also include information about patient characteristics and the treatment performed, including cancer clinical stage, intention and lines of therapy, primary sites, drugs requested, date of chemotherapy started, and expected finishing date. After the anonymization process, SIA data are also publicly available and are generally used for activities and epidemiological planning to face cancer in Brazil. SIA is updated monthly, with an estimated delay of 2 months.

### 4.2. Participants and Data Collection

We included COVID-19 patients admitted, between 1 January 2020, and 28 September 2020, to any Brazilian hospitals registered as cancer centers or academic hospitals (i.e., University hospitals). We selected only patients with a positive SARS-CoV-2 reverse transcriptase-polymerase chain reaction (RT-PCR) assay of nasal or pharyngeal swab specimens. Patients with only serologic and/or epidemiological criteria for COVID-19 diagnosis were excluded from the analysis. We also excluded COVID-19 patients treated in an outpatient setting, patients younger than 18 years, pregnant and puerperal women, and patients with missing information in hospitalization definition. To warrant data quality, we excluded patients without reported X-ray information. We also excluded patients currently receiving inpatient care; thus, we assumed either hospital discharge or death as possible outcomes.

Patients with defined cancer in the SIVEP dataset were actively searched using a probabilistic linkage in SIA dataset. We used all available records in all Brazilian territories, from January 2019 to September 2020, the last available record in the SIA. Birthdate, sex, city of residence, institution, and cancer primary site were used to match. All pairs were revised for integrity and excluded if inconsistent.

### 4.3. Variables Analyzed

We collected the following variables from the SIVEP registry: Age, sex, type of presentation (critical versus non-critical), and comorbidities (cardiovascular diseases, nephropathy, chronic lung disease, diabetes, cancer, and neurological disease). Cardiovascular disease was defined as the presence of hypertension or any heart disease. Chronic lung disease was defined as chronic obstructive pulmonary disease or interstitial lung disease (but not asthma). Neurological disease was defined as inflammatory or neurodegenerative diseases and vascular diseases of the central nervous system. The SIVEP electronic form does not have a dedicated blank for cancer definition, but the reporting health professional may specify the presence of cancer in a free text area. We searched for a cancer description in this free area. Cancer was defined as any oncological disease reported in SIVEP. Critical presentation was defined as the use of invasive mechanical ventilation. Reporting clinical data is non-mandatory in SIVEP, so many variables were not reported for some patients. When a variable was not reported, we considered it as negative (absence of disease or did not undergo mechanical ventilation), provided that the case under analysis fulfilled the selection criteria. We predicted a possible reporting of cancer as a form of immune depression (which, unlike cancer, has a dedicated blank in SIVEP’s electronic form). Some professionals may assign a cancer patient as “positive” for immune suppression, and at the same time not fill “cancer” in the free text space; thus, we excluded patients with this condition to refine non-cancer patients, assuming we would lose some patients.

From the SIA, we extracted information about the tumor primary site, clinical stage, modality of treatment, drugs delivered, and expected dates of completion of treatment. All tumor sites were classified according to the International Classification of Diseases (ICD-10), and staged according to American Joint Committee on Cancer (AJCC) criteria, where feasible. In Brazil, the drugs requested for treatment follow national patterns of cancer treatment by the Brazilian Public Health System. In SIA, clinical oncologists describe the chemotherapy scheme requested. We assumed that the treatment requested was delivered. All patients undergoing active treatment have their Autorização de Procedimento de Alta Complexidade (APAC—authorization for execution of high complexity procedure) renewed monthly, so those presenting clinical symptoms of COVID-19 infection until 30 days of the expected completion date were defined as in active treatment, while those outside this range were treated as non-active treatment.

Cancer stage was grouped as “I to II”, “III to IV”, “unknown” (i.e., TxNxMx), and “others”, with the latter referring to neoplasms with a nonstandard AJCC clinical stage protocol (e.g., lymphoma). The line of treatment was grouped into palliative first line, palliative second line, hematological chemotherapy schemes, and neoadjuvant/definitive/adjuvant settings. We also classified according to single or non-single (doublets/triplets) chemotherapy regimens modalities. The primary sites were defined as head and neck, gastrointestinal, lung, breast, gynecological, prostate, central nervous system, hematological, and others. Treatments were classified regarding the presence of anti-metabolites, microtubule inhibitors, alkylating agents, cisplatin, topoisomerase 1 inhibitors, and topoisomerase 2 inhibitors.

### 4.4. Analysis

Initially, we compared patients under chemotherapy (active and non-active) against a pair-matched non-oncological control group. The matching process was performed according to the propensity score associated with baseline variables (age, sex, race, Brazilian region, type of presentation, and comorbidities) in both eligible controls and cases. Then, to compare the effect of the active chemotherapy per se we built a second propensity score matching between patients in active chemotherapy and in non-active chemotherapy. We added oncological variables in this step (clinical stage, regimen, anti-metabolic, microtubule inhibitor, alkylating, cisplatin, topoisomerase 1 inhibitor, topoisomerase 2 inhibitor, line of treatment, and the primary site).

To further explore the effect of drug classes per se, we built propensity score pair matching comparing patients in the active chemotherapy group and the use of anti-metabolic, microtubule inhibitor, alkylating, cisplatin, topoisomerase 1 inhibitor, and topoisomerase 2 inhibitor against the overall non-active chemotherapy group. In this step, the drugs class variables were excluded from the propensity score model.

All propensity score models used a 1:1 (one case to one control) proportion for pair matching. All the variables were accessed using an optimal algorithm for propensity score pair matching, and only observation with propensity scores in the common support region were selected for matching, without a limiting caliper.

Cases and controls were compared before and after the matching process, with a chi-square test to examine the matching performance; we also verified the standardized mean logit propensity score difference before and after the matching process in all variables inside the model. Outcomes after matching were carried out with univariate conditional logistic regression and graphically represented as a cumulative incidence of events (death), accounted since the date of hospitalization, or the time of the start of symptoms, if unavailable.

To analyze the risk factors associated with death in patients with COVID-19 infection and under cytotoxic chemotherapy in a classical multivariate model, we first ran a univariate model. Then, we performed a multivariate logistic regression, with backward elimination, keeping in the final model variables with significance superior to *p* < 0.10. We previously planned to do exploratory analyses to further investigate the risk factors found. All the data processing and analysis were done in SAS 9.4 software (SAS Institute Inc, Cary, United States of America).

The research was previously submitted and accepted at Universidade Estadual de Campinas institution review board, with a waiver of informed consent (Certificado de Apresentação de Apreciação Ética (CAAE): 31591820.5.0000.5404).

## 5. Conclusions

Our data shows that cancer patients with past or present chemotherapy are at increased risk of death from COVID-19. This risk appears to be associated with the cancer itself, since active chemotherapy did not lead to an increased risk compared to past chemotherapy. We also noted a protective effect of topoisomerase II inhibitor and alkylating agents use, which warrants further investigation.

## Figures and Tables

**Table 1:** Baseline variables before and after active and non-active chemotherapy groups propensity score matching.

Characteristic	Non-Matched			Pair Matched			Standardized Mean Difference		
	Active Chemo. *n* = 431	Non-Active Chemo. *n* = 250	*p*	Active Chemo. *n* = 246	Non-Active Chemo. *n* = 246	*p*	All	Region	Matched
Age			0.0182			0.9893			
<50 years	121 (28.1)	46 (18.4)		49 (19.9)	46 (18.7)		0.23058	0.19103	0.02907
50 to 64 years	172 (39.9)	102 (40.8)		99 (40.2)	101 (41.1)		−0.01820	−0.00805	−0.01657
65 to 79 years	122 (28.3)	93 (37.2)		89 (36.2)	90 (36.6)		−0.19036	−0.16575	−0.00870
≥80 years	16 (3.7)	9 (3.6)		9 (3.7)	9 (3.7)		0.00598	0.00661	0.00000
Sex			0.1849			0.7183	−0.10541	−0.10407	0.03268
Male	186 (43.2)	121 (48.4)		122 (49.6)	118 (48)				
Female	245 (56.8)	129 (51.6)		124 (50.4)	128 (52)				
Comorbidities									
Heart Disease	83 (19.3)	60 (24)	0.1430	60 (24.4)	57 (23.2)	0.7507	−0.11538	−0.08634	0.02967
Diabetes	66 (15.3)	38 (15.2)	0.9684	38 (15.5)	38 (15.5)	1.0000	0.00315	−0.00225	0.00000
Neurologic disease	5 (1.2)	8 (3.2)	0.0608	5 (2)	6 (2.4)	0.7604	−0.14003	−0.08629	−0.02790
Chronic lung disease	17 (3.9)	12 (4.8)	0.5940	11 (4.5)	12 (4.9)	0.8309	−0.04186	−0.04203	−0.01988
Nephropathy	23 (5.3)	16 (6.4)	0.5648	15 (6.1)	15 (6.1)	1.0000	−0.04526	−0.02810	0.00000
Critical Presentation	97 (22.5)	61 (24.4)	0.5724	59 (24)	61 (24.8)	0.8337	−0.04472	−0.05520	−0.01919
Southeast	281 (65.2)	138 (55.2)	0.0097	138 (56.1)	137 (55.7)	0.9277	0.20531	0.18656	0.00835
Clinical Stage			0.0747			0.9835			
I/II	64 (14.9)	42 (16.8)		41 (16.7)	42 (17.1)		0.01846	0.02824	−0.03477
III/IV	272 (63.1)	172 (68.8)		170 (69.1)	168 (68.3)		0.12211	0.09351	0.00000
Unknown	27 (6.3)	7 (2.8)		8 (3.3)	7 (2.9)		−0.16690	−0.14763	−0.01958
Other	68 (15.8)	29 (11.6)		27 (11)	29 (11.8)		−0.12157	−0.09036	0.02366
Mult. Drug Regimen	237 (55)	132 (52.8)	0.5806	135 (54.9)	130 (52.9)	0.6511	−0.04391	−0.03066	−0.04078
Drug Class									
Anti-Metabolic	169 (39.2)	96 (38.4)	0.8342	97 (39.4)	94 (38.2)	0.7814	0.01665	0.00663	0.02503
Microtubule inhib.	153 (35.5)	86 (34.4)	0.7721	82 (33.3)	84 (34.2)	0.8488	0.02305	0.01766	−0.01705
Alkylating	115 (26.7)	53 (21.2)	0.1097	56 (22.8)	53 (21.5)	0.7447	0.12874	0.11029	0.02864
Cisplatin	186 (43.2)	127 (50.8)	0.0537	127 (51.6)	125 (50.8)	0.8568	−0.15362	−0.14223	0.01634
Type 1 topois. inhib.	36 (8.4)	10 (4)	0.0291	13 (5.3)	10 (4.1)	0.5217	0.18156	0.11641	0.05087
Type 2 topois. inhib.	94 (21.8)	40 (16)	0.0660	40 (16.3)	40 (16.3)	1.0000	0.14879	0.11031	0.00000
Treatment			0.0077			0.4682			
Palliative 1st Line	193 (44.8)	105 (42)		119 (48.4)	103 (41.9)		−0.05602	−0.07571	−0.13108
Palliative 2nd Line	36 (8.4)	22 (8.8)		16 (6.5)	22 (8.9)		0.01595	0.02385	0.08697
Hematological cancer	111 (25.8)	44 (17.6)		41 (16.7)	44 (17.9)		−0.25943	−0.21717	−0.02354
Neoadj./Definit./Adjuv.	91 (21.1)	79 (31.6)		70 (28.5)	77 (31.3)		0.23933	0.22339	0.06494
Primary Site			0.0694			0.9995			
GI	121 (28.1)	78 (31.2)		83 (33.7)	78 (31.7)		−0.06849	−0.08101	0.00894
Head and Neck	18 (4.2)	17 (6.8)		15 (6.1)	16 (6.5)		−0.11539	−0.10312	−0.01795
Lung	30 (7)	21 (8.4)		17 (6.9)	21 (8.5)		−0.05408	−0.04458	−0.04600
Others	15 (3.5)	14 (5.6)		14 (5.7)	12 (4.9)		−0.10195	−0.06780	0.00000
Breast	66 (15.3)	24 (9.6)		25 (10.2)	24 (9.8)		0.17366	0.16172	0.06203
Gynecological	42 (9.7)	33 (13.2)		33 (13.4)	32 (13)		−0.10858	−0.10351	−0.02565
Prostate	9 (2.1)	8 (3.2)		7 (2.9)	8 (3.3)		−0.06934	−0.07311	0.00000
Cent. Nervous Syst.	8 (1.9)	4 (1.6)		4 (1.6)	4 (1.6)		0.01966	0.01748	0.00000
Hematologic	111 (25.8)	44 (17.6)		41 (16.7)	44 (17.9)		0.19887	0.19157	0.00995
Sarcomas	11 (2.6)	7 (2.8)		7 (2.9)	7 (2.9)		−0.01535	−0.01853	−0.02529
Logit Prop. Score							0.57261	0.48064	0.06873
Outcome			0.0141			0.3322			
Hospital Discharge	166 (38.5)	73 (29.2)		83 (33.9)	73 (29.8)				
Death	265 (61.5)	177 (70.8)		162 (66.1)	172 (70.2)				

Abbreviations: Adjuv: Adjuvant; Cent.: Central; Definit: Definitive; GI: Gastrointestinal; Inhib: Inhibitor; Mult.: Multiple; Neoadj: Neoadjuvant; Prop: Propensity; Syst: System; Topois: Topoisomerase.

**Table 2:** Univariate and multivariate analysis of risk to death in COVID-19 patients.

Characteristic	Events/Number at Risk	Univariate Analysis		Multivariate Analysis	
		OR (95% CI)	*p*	OR (95% CI)	*p*
Age					
<65 years	277/441	Reference (A)			
≥65 years	165/240	1.3 (0.93–1.82)	0.1213		
Sex					
Male	206/307	Reference (B)			
Female	236/374	0.84 (0.61–1.15)	0.2768		
Comorbidities					
Heart Disease	101/143	1.39 (0.93–2.07)	0.1075		
Diabetes	70/104	1.14 (0.73–1.77)	0.5771		
Neurologic disease	9/13	1.22 (0.37–4.01)	0.7418		
Chronic lung disease	19/29	1.03 (0.47–2.25)	0.9439		
Nephropathy	29/39	1.61 (0.77–3.36)	0.2066		
Southeast Brazilian Region	261/419	0.74 (0.53–1.03)	0.0712	0.74 (0.53–1.03)	0.0777
Clinical Stage					
I/II	61/106	Reference (C)			
III/IV	293/444	1.43 (0.93–2.21)	0.1039		
Unknown	21/34	1.19 (0.54–2.63)	0.6642		
Other	67/97	1.65 (0.93–2.94)	0.0903		
Mult. Drug Regimen	238/369	0.96 (0.7–1.32)	0.8094		
Drug Class					
Anti-Metabolic	175/265	1.09 (0.79–1.5)	0.6210		
Microtubule inhibitor	154/239	0.97 (0.7–1.35)	0.8501		
Alkylating	95/168	0.62 (0.44–0.89)	0.0092		
Cisplatin	216/313	1.4 (1.02–1.92)	0.0388		
Type 1 topois. inhib.	29/46	0.92 (0.49–1.71)	0.7842		
Type 2 topois. inhib.	69/134	0.5 (0.34–0.73)	0.0003	0.55 (0.36–0.85)	0.0070
Chemotherapy setting					
Neoadj./Definit./Adjuv.	94/170	Reference (D)			
Palliative 1st Line	209/298	1.9 (1.28–2.81)	0.0013	1.83 (1.22–2.74)	0.0038
Palliative 2nd Line	43/58	2.32 (1.2–4.49)	0.0127	2.26 (1.15–4.45)	0.0180
Hematological cancer	96/155	1.32 (0.84–2.05)	0.2253	1.68 (1.05–2.7)	0.0310
Primary Site					
GI	126/199	Reference (E)			
Head and Neck	18/35	0.61 (0.3–1.26)	0.1852		
Lung	38/51	1.69 (0.85–3.39)	0.1360		
Others	25/29	3.62 (1.21–10.81)	0.0212		
Breast	51/90	0.76 (0.46–1.26)	0.2832		
Gynecological	55/75	1.59 (0.89–2.87)	0.1201		
Prostate	13/17	1.88 (0.59–5.99)	0.2838		
Cent. Nervous Syst.	7/12	0.81 (0.25–2.65)	0.7288		
Hematologic	96/155	0.94 (0.61–1.46)	0.7898		
Sarcomas	13/18	1.51 (0.52–4.4)	0.4534		
Active chemotherapy	265/431	0.66 (0.47–0.92)	0.0144	0.66 (0.46–0.93)	0.0169

Abbreviations: Adjuv: Adjuvant; Definit: Definitive; GI: Gastrointestinal; Inhib: Inhibitor; Mult.: Multiple; Neoadj: Neoadjuvant; Topois: Topoisomerase. (**A**) Patients with less than 65 years were used as reference to calculate the OR for patients with more than 65 years (characteristic: Age), (**B**): Male patients were used as reference to calculate the OR for female patients (characteristic: Sex), (**C**): Patients with clinical stage I/II were used as reference to calculate the OR for III/IV, unknown and others patients (characteristic: Clinical stage), (**D**): Neoadj./Definit./Adjuv. were used as reference to calculate the OR for Palliative 1st Line, Palliative 2nd Line and Hematological cancer (characteristic: Chemotherapy objective), (**E**): Patients with GI cancers were used as reference to calculate the OR for head and neck, lung, others, breast gynecological, prostate, central nervous system, hematologic and sarcomas (characteristic: Primary site).

## Supplementary Materials

The following are available online at https://www.mdpi.com/2072-6694/12/12/3490/s1, Figure S1: Standardized Mean Differences in Active versus non−Active chemotherapy Propensity Score, Figure S2: Cumulative Distribution of Logit Propensity Score in Active versus non−Active Chemotherapy Propensity Score, Figure S3: Standardized Mean Differences in Chemotherapy versus non−Cancer Controls Propensity Score, Figure S4: Standardized Mean Differences in Active Anti−Metabolic versus non−Active chemotherapy Propensity Score, Figure S5: Standardized Mean Differences in Active Microtubule Inhibitor versus non−Active chemotherapy Propensity Score, Figure S6: Standardized Mean Differences in Active Alkylating versus non−Active chemotherapy Propensity Score, Figure S7: Standardized Mean Differences in Active Cisplatin versus non−Active chemotherapy Propensity Score, Figure S8: Standardized Mean Differences in Active Topoisomerase I Inhibitor versus non−Active chemotherapy Propensity Score, Figure S9: Standardized Mean Differences in Active Topoisomerase II inhibitor versus non−Active chemotherapy Propensity Score, Table S1: Baseline variables before and after active and non−active chemotherapy and non−cancer groups propensity score matching, Table S2: Distribution of all cohort according to detailed Primary Site, Table S3: Baseline variables according to Primary Site, Table S4: Baseline variables before and after active chemotherapy (anti−metabolic) use and non−active chemotherapy groups propensity score matching, Table S5: Baseline variables before and after active chemotherapy (microtubule inhibitor) use and non−active chemotherapy groups propensity score matching, Table S6: Baseline variables before and after active chemotherapy (alkylating) use and non−active chemotherapy groups propensity score matching, Table S7: Baseline variables before and after active chemotherapy (cisplatin) use and non−active chemotherapy groups propensity score matching, Table S8: Baseline variables before and after active chemotherapy (topoisomerase I inhibitor) use and non−active chemotherapy groups propensity score matching, Table S9: Baseline variables before and after active chemotherapy (topoisomerase II inhibitor) use and non−active chemotherapy groups propensity score matching.
