# Supplementary material for: Outcomes of COVID-19 Patients under Cytotoxic Cancer Chemotherapy in Brazil
Source: Cancers (Basel). 2020 Nov 24;12(12):3490. doi: 10.3390/cancers12123490 (PMC7760330; doi:10.3390/cancers12123490)
Supplement: Supplementary file 1 [file cancers-12-03490-s001.pdf]

Supplemental Materials: Outcomes of COVID-19 Patients under Cytotoxic Cancer Chemotherapy in Brazil

Mateus Bringel Oliveira Duarte, Frederico Leal, Juliana Luz Passos Argenton and José Barreto Campello Carvalheira

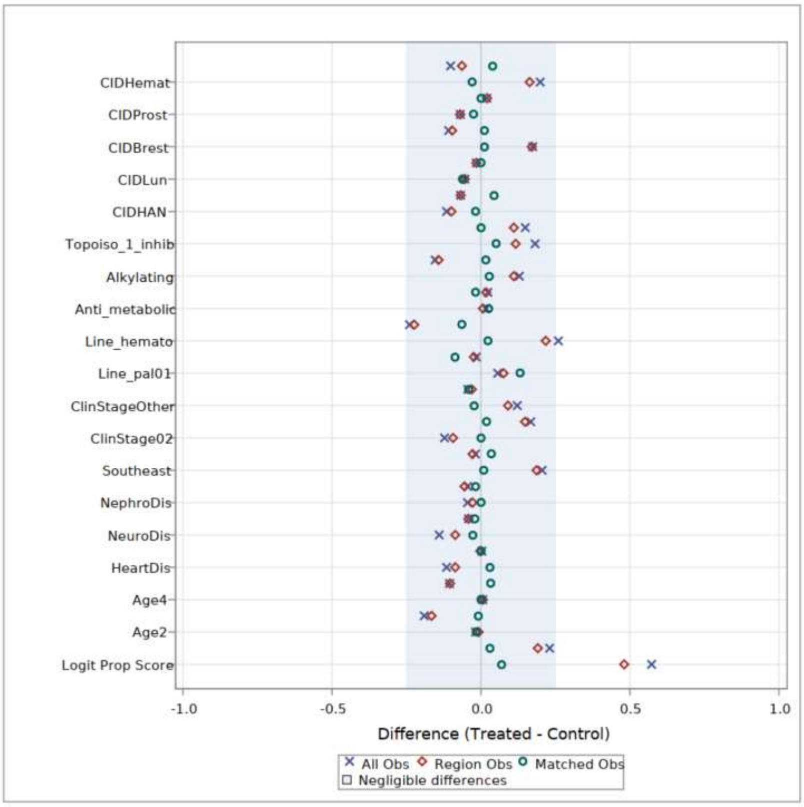

Figure S1. Standardized Mean Differences in Active versus non−Active chemotherapy Propensity Score. Abbreviations: CIDOther: Primary site: Other; CIDHemat: Primary Site: Hematologic; CIDProst: Primary Site: Prostate; CIDGine: Primary Site Gynecological; CIDBrest: Primary Site Breast; CIDSar: Primary Site Sarcomas; CIDLun: Primary Site Lung; CIDGI: Primary Site Gastrointestinal; CIDHAN: Primary Site Head and Neck; Line_neo_adj: Neoadjuvant, Definitive or Adjuvant Line of Treatment; Line_hemato: Hematologic Line of Treatment; Line_pal02: 2nd Line of Treatment; Line_pal01: 1st Line of Treatment; ClinStageOther: Other Clinical Stage; ClinStageUnknown: Unknown Clinical Stage; ClinStage02: III/IV Clinical Stage; ClinStage01: I/II Clinical Stage; : Age1: Less than 50 years; Age2: 50 to 64 years; Age3: 65 to 79 years; Age 4: Greater or equal 80 years.

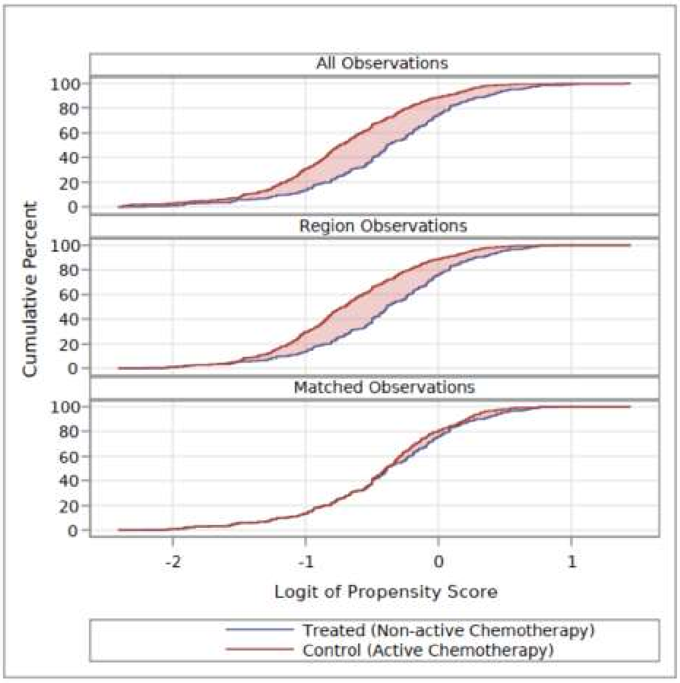

Figure S2. Cumulative Distribution of Logit Propensity Score in Active versus non−Active Chemotherapy Propensity Score.

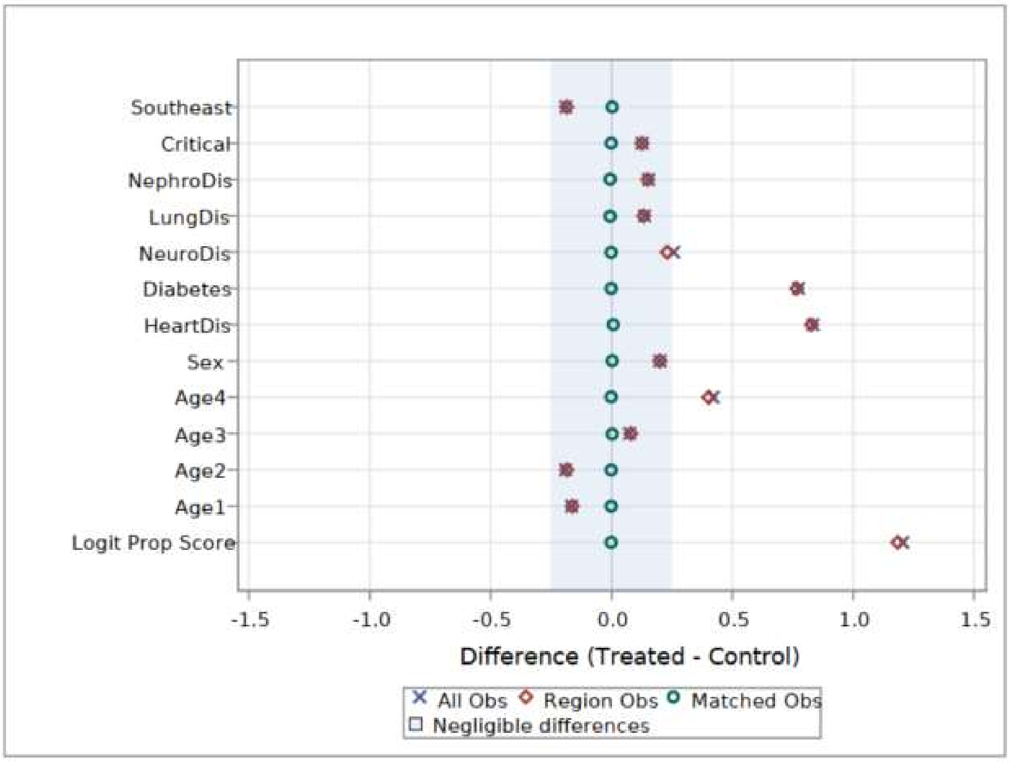

Figure S3. Standardized Mean Differences in Chemotherapy versus non−Cancer Controls Propensity Score. Abbreviations: Age1: Less than 50 years; Age2: 50 to 64 years; Age3: 65 to 79 years; Age 4: Greater or equal 80 years.

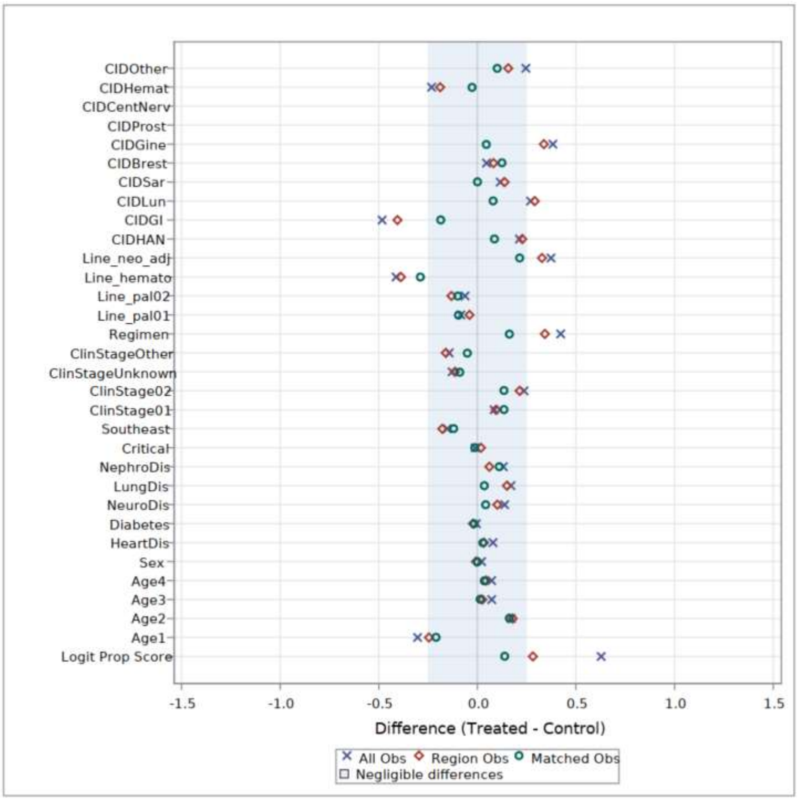

Figure S4. Standardized Mean Differences in Active Anti−Metabolic versus non−Active chemotherapy Propensity Score. Abbreviations: CIDOther: Primary site: Other; CIDHemat: Primary Site: Hematologic; CIDProst: Primary Site: Prostate; CIDGine: Primary Site Gynecological; CIDBrest: Primary Site Breast; CIDSar: Primary Site Sarcomas; CIDLun: Primary Site Lung; CIDGI: Primary Site Gastrointestinal; CIDHAN: Primary Site Head and Neck; Line_neo_adj: Neoadjuvant, Definitive or Adjuvant Line of Treatment; Line_hemato: Hematologic Line of Treatment; Line_pal02: 2nd Line of Treatment; Line_pal01: 1st Line of Treatment; ClinStageOther: Other Clinical Stage; ClinStageUnknown: Unknown Clinical Stage; ClinStage02: III/IV Clinical Stage; ClinStage01: I/II Clinical Stage; Age1: Less than 50 years; Age2: 50 to 64 years; Age3: 65 to 79 years; Age 4: Greater or equal 80 years.

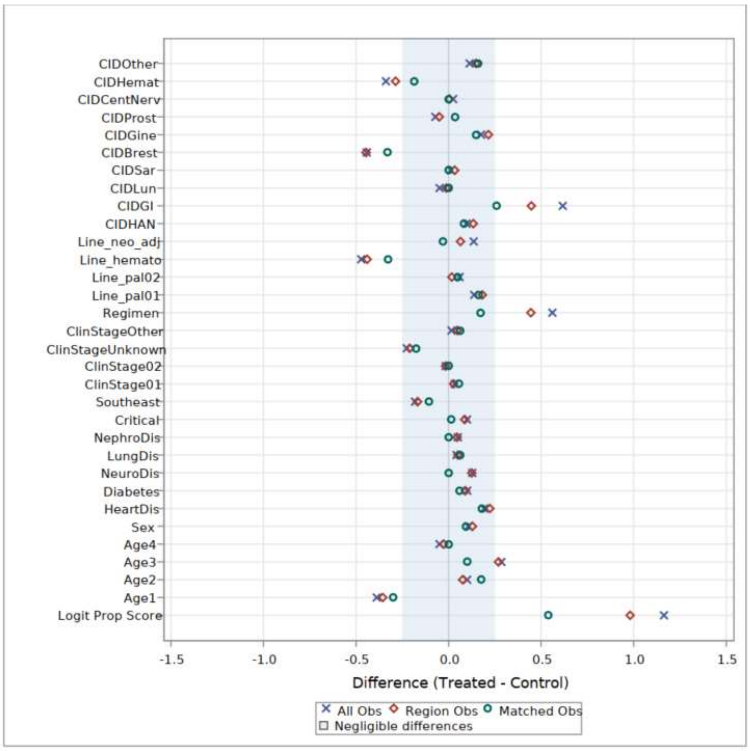

Figure S5. Standardized Mean Differences in Active Microtubule Inhibitor versus non−Active chemotherapy Propensity Score. Abbreviations: CIDOther: Primary site: Other; CIDHemat: Primary Site: Hematologic; CIDProst: Primary Site: Prostate; CIDGine: Primary Site Gynecological; CIDBrest: Primary Site Breast; CIDSar: Primary Site Sarcomas; CIDLun: Primary Site Lung; CIDGI: Primary Site Gastrointestinal; CIDHAN: Primary Site Head and Neck; Line_neo_adj: Neoadjuvant, Definitive or Adjuvant Line of Treatment; Line_hemato: Hematologic Line of Treatment; Line_pal02: 2nd Line of Treatment; Line_pal01: 1st Line of Treatment; ClinStageOther: Other Clinical Stage; ClinStageUnknown: Unknown Clinical Stage; ClinStage02: III/IV Clinical Stage; ClinStage01: I/II Clinical Stage; Age1: Less than 50 years; Age2: 50 to 64 years; Age3: 65 to 79 years; Age 4: Greater or equal 80 years.

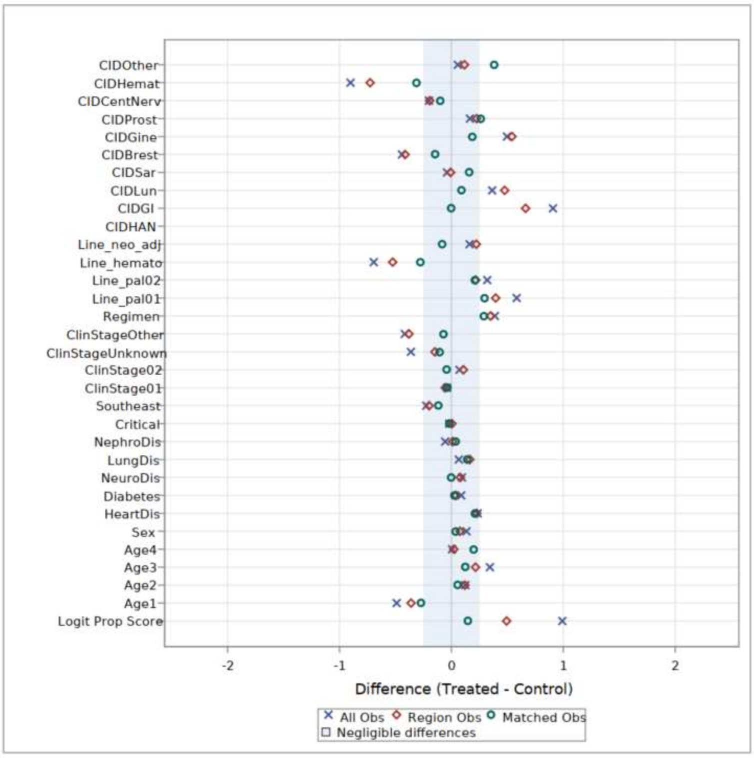

Figure S6. Standardized Mean Differences in Active Alkylating versus non−Active chemotherapy Propensity Score. Abbreviations: CIDOther: Primary site: Other; CIDHemat: Primary Site: Hematologic; CIDProst: Primary Site: Prostate; CIDGine: Primary Site Gynecological; CIDBrest: Primary Site Breast; CIDSar: Primary Site Sarcomas; CIDLun: Primary Site Lung; CIDGI: Primary Site Gastrointestinal; CIDHAN: Primary Site Head and Neck; Line_neo_adj: Neoadjuvant, Definitive or Adjuvant Line of Treatment; Line_hemato: Hematologic Line of Treatment; Line_pal02: 2nd Line of Treatment; Line_pal01: 1st Line of Treatment; ClinStageOther: Other Clinical Stage; ClinStageUnknown: Unknown Clinical Stage; ClinStage02: III/IV Clinical Stage; ClinStage01: I/II Clinical Stage; Age1: Less than 50 years; Age2: 50 to 64 years; Age3: 65 to 79 years; Age 4: Greater or equal 80 years.

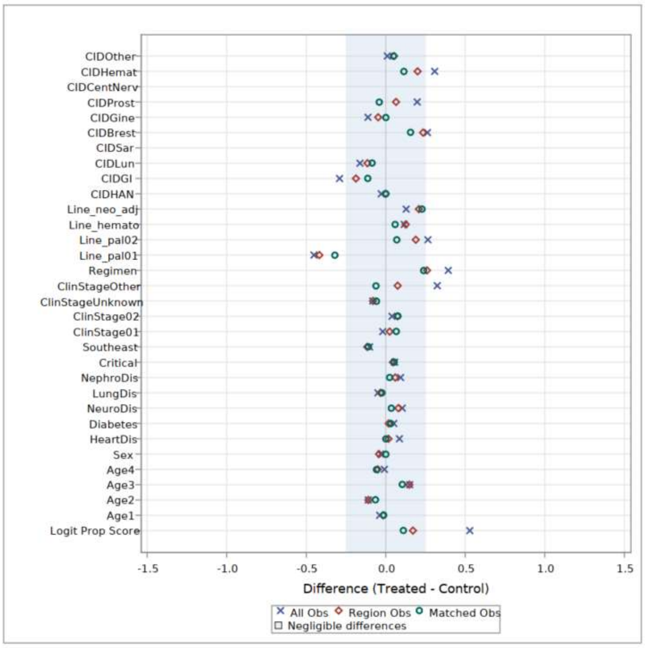

Figure S7. Standardized Mean Differences in Active Cisplatin versus non−Active chemotherapy Propensity Score. Abbreviations: CIDOther: Primary site: Other; CIDHemat: Primary Site: Hematologic; CIDProst: Primary Site: Prostate; CIDGine: Primary Site Gynecological; CIDBrest: Primary Site Breast; CIDSar: Primary Site Sarcomas; CIDLun: Primary Site Lung; CIDGI: Primary Site Gastrointestinal; CIDHAN: Primary Site Head and Neck; Line_neo_adj: Neoadjuvant, Definitive or Adjuvant Line of Treatment; Line_hemato: Hematologic Line of Treatment; Line_pal02: 2nd Line of Treatment; Line_pal01: 1st Line of Treatment; ClinStageOther: Other Clinical Stage; ClinStageUnknown: Unknown Clinical Stage; ClinStage02: III/IV Clinical Stage; ClinStage01: I/II Clinical Stage; Age1: Less than 50 years; Age2: 50 to 64 years; Age3: 65 to 79 years; Age 4: Greater or equal 80 years.

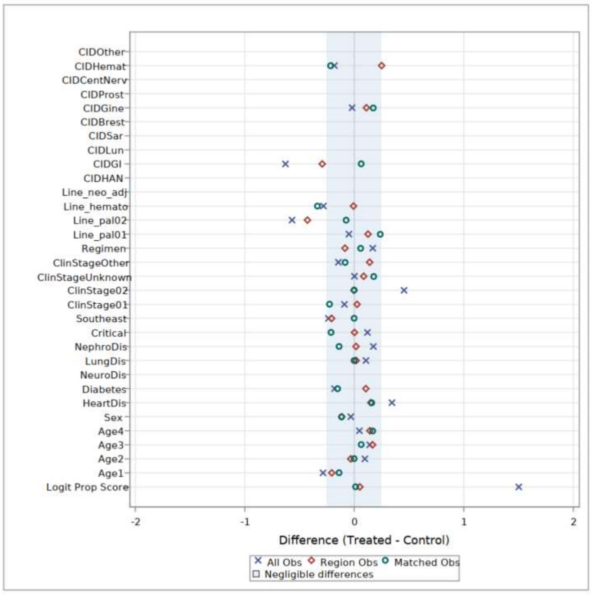

Figure S8. Standardized Mean Differences in Active Topoisomerase I Inhibitor versus non−Active chemotherapy Propensity Score. Abbreviations: CIDOther: Primary site: Other; CIDHemat: Primary Site: Hematologic; CIDProst: Primary Site: Prostate; CIDGine: Primary Site Gynecological; CIDBrest: Primary Site Breast; CIDSar: Primary Site Sarcomas; CIDLun: Primary Site Lung; CIDGI: Primary Site Gastrointestinal; CIDHAN: Primary Site Head and Neck; Line_neo_adj: Neoadjuvant, Definitive or Adjuvant Line of Treatment; Line_hemato: Hematologic Line of Treatment; Line_pal02: 2nd Line of Treatment; Line_pal01: 1st Line of Treatment; ClinStageOther: Other Clinical Stage; ClinStageUnknown: Unknown Clinical Stage; ClinStage02: III/IV Clinical Stage; ClinStage01: I/II Clinical Stage; Age1: Less than 50 years; Age2: 50 to 64 years; Age3: 65 to 79 years; Age 4: Greater or equal 80 years.

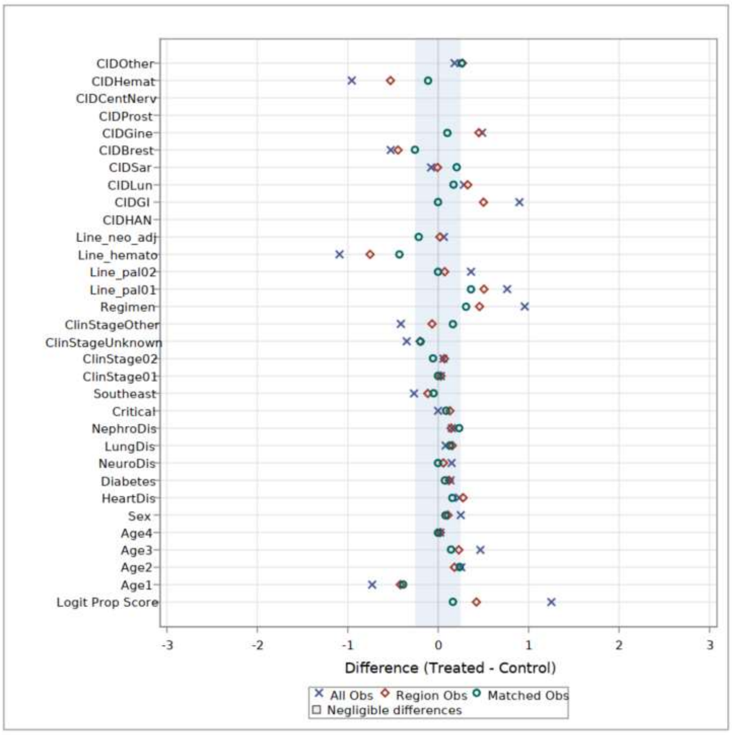

Figure S9. Standardized Mean Differences in Active Topoisomerase II inhibitor versus non−Active chemotherapy Propensity Score. Abbreviations: CIDOther: Primary site: Other; CIDHemat: Primary Site: Hematologic; CIDProst: Primary Site: Prostate; CIDGine: Primary Site Gynecological; CIDBrest: Primary Site Breast; CIDSar: Primary Site Sarcomas; CIDLun: Primary Site Lung; CIDGI: Primary Site Gastrointestinal; CIDHAN: Primary Site Head and Neck; Line_neo_adj: Neoadjuvant, Definitive or Adjuvant Line of Treatment; Line_hemato: Hematologic Line of Treatment; Line_pal02: 2nd Line of Treatment; Line_pal01: 1st Line of Treatment; ClinStageOther: Other Clinical Stage; ClinStageUnknown: Unknown Clinical Stage; ClinStage02: III/IV Clinical Stage; ClinStage01: I/II Clinical Stage; Age1: Less than 50 years; Age2: 50 to 64 years; Age3: 65 to 79 years; Age 4: Greater or equal 80 years.

Table S1. Baseline variables before and after active and non−active chemotherapy and non−cancer groups propensity score matching.

	**Non−Matched**			**Pair Matched**			**Standardized Mean Difference**		
**Characteristic**	**Active and Non−Active Chemo. *n* = 681**	**Non−Cancer *n* = 38468**	***p***	**Active and Non−Active Chemo. *n* = 681**	**Non−Cancer *n* = 38,468**	***p***	**All**	**Region**	**Matched**
Age			<0.0001			0.9999			
<50 years	167 (24.5)	10192 (26.5)		167 (24.5)	166 (24.4)		−0.16456	−0.16198	−0.00361
50 to 64 years	274 (40.2)	11730 (30.5)		274 (40.2)	274 (40.2)		−0.18970	−0.18552	0.00000
65 to 79 years	215 (31.6)	11379 (29.6)		215 (31.6)	216 (31.7)		0.07492	0.07959	0.00312
≥80 years	25 (3.7)	5167 (13.4)		25 (3.7)	25 (3.7)		0.42036	0.40231	0.00000
Sex			<0.0001			0.9566	0.19992	0.19982	0.00295
Male	307 (45.1)	22118 (57.5)		307 (45.1)	308 (45.2)				
Female	374 (54.9)	16350 (42.5)		374 (54.9)	373 (54.8)				

Comorbidities									
Heart Disease	143 (21)	15866 (41.2)	<0.0001	143 (21)	145 (21.3)	0.8944	0.83356	0.82771	0.00650
Diabetes	104 (15.3)	11333 (49)	<0.0001	104 (15.3)	103 (15.1)	0.9398	0.77371	0.76632	−0.00337
Neurologic disease	13 (1.9)	2026 (5.3)	<0.0001	13 (1.9)	13 (1.9)	1.0000	0.25685	0.23010	0.00000
Chronic lung disease	29 (4.3)	2152 (5.6)	0.1319	29 (4.3)	28 (4.1)	0.8924	0.13670	0.13317	−0.00627
Nephropathy	39 (5.7)	2630 (6.8)	0.2546	39 (5.7)	38 (5.6)	0.9066	0.15248	0.14945	−0.00550
Critical Presentation	158 (23.2)	9848 (25.6)	0.1548	158 (23.2)	158 (23.2)	1.0000	0.12649	0.12562	0.00000
Southeast	419 (61.5)	19955 (51.9)	<0.0001	419 (61.5)	420 (61.7)	0.9556	−0.18862	−0.18608	0.00298
Logit Prop. Score							1.20518	1.18460	0.00000
Outcome			<0.0001			<.0001			
Hospital Discharge	239 (35.1)	24213 (62.9)		239 (35.1)	464 (68.1)				
Death	442 (64.9)	14255 (37.1)		442 (64.9)	217 (31.9)				

Table S2. Distribution of all cohort according to detailed Primary Site.

Primary Site	Active Chemotherapy (*n* = 431)	Non−Active Chemotherapy (*n* = 250)	Total (*n* = 681)
Mouth	3 (0.70)	4 (1.60)	7
Oropharynx	7 (1.62)	3 (1.20)	10
Nasopharynx	0 (0.00)	3 (1.20)	3
Other Head and Neck	1 (0.23)	0 (0.00)	1
Esophagus	20 (4.64)	9 (3.60)	29
Stomach	19 (4.41)	10 (4.00)	29
Colorectal	58 (13.46)	52 (20.80)	110
Anus	6 (1.39)	2 (0.80)	8
Hepatic and biliary tree	7 (1.62)	1 (0.40)	8
Pancreas	11 (2.55)	4 (1.60)	15
Larynx	5 (1.16)	7 (2.80)	12
Lung	30 (6.96)	21 (8.40)	51
Bones	3 (0.70)	1 (0.40)	4
Skin melanoma	5 (1.16)	1 (0.40)	6
Sarcomas	7 (1.62)	5 (2.00)	12
Breast	66 (15.31)	24 (9.60)	90
Cervix	30 (6.96)	17 (6.80)	47
Uterine	4 (0.93)	2 (0.80)	6
Ovaries	8 (1.86)	14 (5.60)	22
Penis	1 (0.23)	0 (0.00)	1
Prostate	9 (2.09)	8 (3.20)	17
Kidney	0 (0.00)	1 (0.40)	1
Bladder	1 (0.23)	8 (3.20)	9
Brain	6 (1.39)	3 (1.20)	9
Medulla	2 (0.46)	1 (0.40)	3
Endocrine glands	0 (0.00)	1 (0.40)	1
Hodgkin lymphoma	6 (1.39)	2 (0.80)	8
non−Hodgkin lymphoma	52 (12.06)	20 (8.00)	72
Myeloma	17 (3.94)	8 (3.20)	25
Leukemias	36 (8.35)	13 (5.20)	49
Other	11 (2.55)	5 (2.00)	16

Table S3. Baseline variables according to Primary Site.

Characteristic	Head And Neck	Gastro−Intestinal	Others	Lung	Sarcoma	Breast	Gyneco−Logical	Prostate	Central Nervous System	Haema−Tologic
Age										
<50 years	5 (14.3)	29 (14.6)	3 (10.3)	1 (2)	11 (61.1)	35 (38.9)	26 (34.7)	0 (0)	4 (33.3)	53 (34.2)
50 to 64 years	21 (60)	91 (45.7)	13 (44.8)	24 (47.1)	6 (33.3)	35 (38.9)	26 (34.7)	4 (23.5)	6 (50)	48 (31)
65 to 79 years	9 (25.7)	76 (38.2)	11 (37.9)	22 (43.1)	1 (5.6)	17 (18.9)	21 (28)	10 (58.8)	2 (16.7)	46 (29.7)
≥80 years	0 (0)	3 (1.5)	2 (6.9)	4 (7.8)	0 (0)	3 (3.3)	2 (2.7)	3 (17.7)	0 (0)	8 (5.2)
Sex										
Male	28 (80)	110 (55.3)	19 (65.5)	37 (72.6)	11 (61.1)	2 (2.2)	0 (0)	17 (100)	5 (41.7)	78 (50.3)
Female	7 (20)	89 (44.7)	10 (34.5)	14 (27.5)	7 (38.9)	88 (97.8)	75 (100)	0 (0)	7 (58.3)	77 (49.7)
Comorbidities										
Heart Disease	6 (17.1)	39 (19.6)	9 (31)	12 (23.5)	3 (16.7)	13 (14.4)	18 (24)	8 (47.1)	0 (0)	35 (22.6)
Diabetes	3 (8.6)	34 (17.1)	6 (20.7)	6 (11.8)	2 (11.1)	9 (10)	11 (14.7)	3 (17.7)	3 (25)	27 (17.4)
Neurologic dis.	2 (5.7)	3 (1.5)	0 (0)	2 (3.9)	1 (5.6)	0 (0)	3 (4)	0 (0)	1 (8.3)	1 (0.7)
Chronic lung dis.	3 (8.6)	8 (4)	1 (3.5)	9 (17.7)	0 (0)	1 (1.1)	2 (2.7)	1 (5.9)	0 (0)	4 (2.6)
Nephropathy	0 (0)	11 (5.5)	4 (13.8)	1 (2)	1 (5.6)	2 (2.2)	5 (6.7)	1 (5.9)	0 (0)	14 (9)
Critical Presentation	6 (17.1)	43 (21.6)	4 (13.8)	11 (21.6)	4 (22.2)	26 (28.9)	24 (32)	1 (5.9)	4 (33.3)	35 (22.6)
Southeast	21 (60)	110 (55.3)	17 (58.6)	26 (51)	10 (55.6)	66 (73.3)	50 (66.7)	14 (82.4)	11 (91.7)	94 (60.7)
Clinical Stage										
I/II	4 (11.4)	37 (18.6)	6 (20.7)	4 (7.8)	1 (5.6)	17 (18.9)	12 (16)	1 (5.9)	0 (0)	24 (15.5)
III/IV	29 (82.9)	156 (78.4)	14 (48.3)	47 (92.2)	17 (94.4)	68 (75.6)	61 (81.3)	15 (88.2)	0 (0)	37 (23.9)
Unknown	2 (5.7)	6 (3)	0 (0)	0 (0)	0 (0)	5 (5.6)	2 (2.7)	1 (5.9)	0 (0)	18 (11.6)
Other	0 (0)	0 (0)	9 (31)	0 (0)	0 (0)	0 (0)	0 (0)	0 (0)	12 (100)	76 (49)
Mult. Drug Reg.	11 (31.4)	127 (63.8)	16 (55.2)	25 (49)	4 (22.2)	49 (54.4)	29 (38.7)	1 (5.9)	3 (25)	104 (67.1)
Drug Class										
Anti−Metabolic	5 (14.3)	156 (78.4)	7 (24.1)	6 (11.8)	2 (11.1)	20 (22.2)	8 (10.7)	0 (0)	0 (0)	61 (39.4)
Microtubule inhib.	12 (34.3)	19 (9.6)	11 (37.9)	27 (52.9)	5 (27.8)	51 (56.7)	29 (38.7)	14 (82.4)	3 (25)	68 (43.9)

Alkylating	0 (0)	1 (0.5)	5 (17.2)	1 (2)	6 (33.3)	45 (50)	1 (1.3)	1 (5.9)	10 (83.3)	98 (63.2)
Cisplatin	29 (82.9)	134 (67.3)	22 (75.9)	37 (72.6)	2 (11.1)	10 (11.1)	58 (77.3)	3 (17.7)	0 (0)	18 (11.6)
Type 1 topois. inhib.	0 (0)	29 (14.6)	0 (0)	0 (0)	0 (0)	0 (0)	5 (6.7)	0 (0)	0 (0)	12 (7.7)
Type 2 topois. inhib.	1 (2.9)	1 (0.5)	2 (6.9)	5 (9.8)	7 (38.9)	40 (44.4)	3 (4)	0 (0)	0 (0)	75 (48.4)
Treatment										
Palliative 1st Line	18 (51.4)	112 (56.3)	22 (75.9)	39 (76.5)	17 (94.4)	28 (31.1)	48 (64)	2 (11.8)	12 (100)	0 (0)
Palliative 2nd Line	0 (0)	23 (11.6)	0 (0)	0 (0)	0 (0)	15 (16.7)	5 (6.7)	15 (88.2)	0 (0)	0 (0)
Hematological cancer	0 (0)	0 (0)	0 (0)	0 (0)	0 (0)	0 (0)	0 (0)	0 (0)	0 (0)	155 (100)
Neoadj./Definit./Adjuv.	17 (48.6)	64 (32.2)	7 (24.1)	12 (23.5)	1 (5.6)	47 (52.2)	22 (29.3)	0 (0)	0 (0)	0 (0)
Active chemotherapy	18 (51.4)	121 (60.8)	15 (51.7)	30 (58.8)	11 (61.1)	66 (73.3)	42 (56)	9 (52.9)	8 (66.7)	111 (71.6)
Outcome										
Hospital Discharge	17 (48.6)	73 (36.7)	4 (13.8)	13 (25.5)	5 (27.8)	39 (43.3)	20 (26.7)	4 (23.5)	5 (41.7)	59 (38.1)
Death	18 (51.4)	126 (63.3)	25 (86.2)	38 (74.5)	13 (72.2)	51 (56.7)	55 (73.3)	13 (76.5)	7 (58.3)	96 (61.9)

Abbreviations: Adjuv.: adjuvant; Definit.: Definitive; Inhib.: Inhibitor; Mult.: Multiple; Neoadj.: Neoadjuvant; Reg.: Regimen; Syst.: System; Topois. Topoisomerase.

Table S4. Baseline variables before and after active chemotherapy (**anti−metabolic**) use and non−active chemotherapy groups propensity score matching.

**Characteristic**	**Non−Matched**			**Pair Matched**			**Standardized Mean Difference**		
	**Active Chemo. *n* = 169**	**Non−Active Chemo. *n* = 250**	***p***	**Active Chemo. *n* = 168**	**Non−Active Chemo. *n* = 168**	***p***	**All**	**Region**	**Matched**
Age			0.0200			0.2591			
< 50 years	53 (31.4)	46 (18.4)		52 (31)	37 (22)		−0.30323	−0.24584	−0.20889
50 to 64 years	55 (32.5)	102 (40.8)		55 (32.7)	68 (40.5)		0.17194	0.17902	0.16116
65 to 79 years	57 (33.7)	93 (37.2)		57 (33.9)	58 (34.5)		0.07263	0.02473	0.01245
≥ 80 years	4 (2.4)	9 (3.6)		4 (2.4)	5 (3)		0.07253	0.04295	0.03501
Sex			0.8309			1.0000	0.02127	−0.00715	0.00000
Male	80 (47.3)	121 (48.4)		79 (47)	79 (47)				
Female	89 (52.7)	129 (51.6)		89 (53)	89 (53)				
Comorbidities									
Heart Disease	35 (20.7)	60 (24)	0.4301	35 (20.8)	37 (22)	0.7903	0.07903	0.03336	0.02860
Diabetes	26 (15.4)	38 (15.2)	0.9589	26 (15.5)	25 (14.9)	0.8792	−0.00513	−0.02249	−0.01654
Neurologic disease	2 (1.2)	8 (3.2)	0.1846	2 (1.2)	3 (1.8)	0.6523	0.13806	0.10106	0.04075
Chronic lung disease	3 (1.8)	12 (4.8)	0.1021	3 (1.8)	4 (2.4)	0.7025	0.17025	0.14965	0.03350
Nephropathy	6 (3.6)	16 (6.4)	0.1995	6 (3.6)	10 (6)	0.3055	0.13134	0.06072	0.10974

Critical Presentation	42 (24.9)	61 (24.4)	0.9160	42 (25)	41 (24.4)	0.8993	−0.01049	0.01805	−0.01382
Southeast	106 (62.7)	138 (55.2)	0.1256	105 (62.5)	95 (56.6)	0.2664	−0.15336	−0.17784	−0.12136
Clinical Stage			0.2407			0.7499			
I/II	26 (15.4)	42 (16.8)		26 (15.5)	31 (18.5)		0.08476	0.09377	0.13577
III/IV	106 (62.7)	172 (68.8)		105 (62.5)	106 (63.1)		0.23662	0.21421	0.13463
Unknown	9 (5.3)	7 (2.8)		9 (5.4)	6 (3.6)		−0.12818	−0.11400	−0.09064
Other	28 (16.6)	29 (11.6)		28 (16.7)	25 (14.9)		−0.14318	−0.16012	−0.05147
Mult. Drug Regime	123 (72.8)	132 (52.8)	<.0001	122 (72.6)	109 (64.9)	0.1260	0.42250	0.34201	0.16362
Treatment			0.0020			0.2036			
Palliative 1st Line	78 (46.2)	105 (42)		78 (46.4)	70 (41.7)		−0.08374	−0.04000	−0.09600
Palliative 2nd Line	18 (10.7)	22 (8.8)		17 (10.1)	12 (7.1)		−0.06250	−0.13158	−0.10049
Hematological cancer	46 (27.2)	44 (17.6)		46 (27.4)	44 (26.2)		−0.41241	−0.38757	−0.28971
Neoadj./Definit./Adjuv.	27 (16)	79 (31.6)		27 (16.1)	42 (25)		0.37327	0.32747	0.21332
Primary Site			<.0001			0.5745			
GI	92 (54.4)	78 (31.2)		91 (54.2)	76 (45.2)		−0.48313	−0.40542	−0.18563
Head and Neck	4 (2.4)	17 (6.8)		4 (2.4)	7 (4.2)		0.21319	0.22747	0.08587
Lung	4 (2.4)	21 (8.4)		4 (2.4)	7 (4.2)		0.26974	0.29097	0.07984
Others	2 (1.2)	14 (5.6)		2 (1.2)	5 (3)		0.24582	0.15638	0.09939
Breast	14 (8.3)	24 (9.6)		14 (8.3)	20 (11.9)		0.04613	0.08179	0.12519
Gynecological	5 (3)	33 (13.2)		5 (3)	7 (4.2)		0.38262	0.33713	0.04448
Prostate	0 (0)	8 (3.2)		0 (0)	0 (0)		.	.	.
Cent. Nervous Syst.	0 (0)	4 (1.6)		0 (0)	0 (0)		.	.	.
Hematologic	46 (27.2)	44 (17.6)		46 (27.4)	44 (26.2)		−0.23223	−0.18893	−0.02874
Sarcomas	2 (1.2)	7 (2.8)		2 (1.2)	2 (1.2)		0.11590	0.13770	0.00000
Logit Prop. Score							0.62757	0.28135	0.13854
Outcome			0.0635			0.1052			
Hospital Discharge	64 (37.9)	73 (29.2)		63 (37.5)	49 (29.2)				
Death	105 (62.1)	177 (70.8)		105 (62.5)	119 (70.8)				

Abbreviations: Adjuv.: adjuvant; Cent.: Central; Definit.: Definitive; GI: Gastrointestinal; Neoadj.: Neoadjuvant; Syst.: System.

Table S5. Baseline variables before and after active chemotherapy (**microtubule inhibitor**) use and non−active chemotherapy groups propensity score matching.

**Characteristic**	**Non−Matched**			**Pair Matched**			**Standardized Mean Difference**		
	**Active Chemo. *n* = 153**	**Non−Active Chemo. *n* = 250**	***p***	**Active Chemo. *n* = 153**	**Non−Active Chemo. *n* = 153**	***p***	**All**	**Region**	**Matched**
Age			0.0008			0.0887			
<50 years	54 (35.3)	46 (18.4)		54 (35.3)	34 (22.2)		−0.38834	−0.35570	−0.30048
50 to 64 years	55 (36)	102 (40.8)		55 (36)	68 (44.4)		0.09991	0.07601	0.17494
65 to 79 years	37 (24.2)	93 (37.2)		37 (24.2)	44 (28.8)		0.28509	0.26946	0.10020

≥80 years	7 (4.6)	9 (3.6)		7 (4.6)	7 (4.6)		−0.04926	−0.02633	0.00000
Sex			0.3039			0.4216	0.10578	0.12889	0.09196
Male	66 (43.1)	121 (48.4)		66 (43.1)	73 (47.7)				
Female	87 (56.9)	129 (51.6)		87 (56.9)	80 (52.3)				
Comorbidities									
Heart Disease	25 (16.3)	60 (24)	0.0674	25 (16.3)	36 (23.5)	0.1155	0.19177	0.22245	0.17999
Diabetes	18 (11.8)	38 (15.2)	0.3333	18 (11.8)	21 (13.7)	0.6071	0.10071	0.09089	0.05748
Neurologic disease	2 (1.3)	8 (3.2)	0.2358	2 (1.3)	2 (1.3)	1.0000	0.12779	0.12463	0.00000
Chronic lung disease	6 (3.9)	12 (4.8)	0.6787	6 (3.9)	8 (5.2)	0.5843	0.04302	0.05061	0.06402
Nephropathy	8 (5.2)	16 (6.4)	0.6297	8 (5.2)	8 (5.2)	1.0000	0.05007	0.04606	0.00000
Critical Presentation	31 (20.3)	61 (24.4)	0.3368	31 (20.3)	32 (20.9)	0.8876	0.09950	0.08685	0.01571
Southeast	98 (64.1)	138 (55.2)	0.0800	98 (64.1)	90 (58.8)	0.3474	−0.18116	−0.16774	−0.10701
Clinical Stage			0.1427			0.3679			
I/II	23 (15)	42 (16.8)		23 (15)	18 (11.8)		0.03351	0.02725	0.05679
III/IV	101 (66)	172 (68.8)		101 (66)	109 (71.2)		−0.01591	−0.01649	0.00000
Unknown	12 (7.8)	7 (2.8)		12 (7.8)	6 (3.9)		−0.22611	−0.21027	−0.17582
Other	17 (11.1)	29 (11.6)		17 (11.1)	20 (13.1)		0.01541	0.04733	0.06180
Mult. Drug Regime	120 (78.4)	132 (52.8)	<.0001	120 (78.4)	108 (70.6)	0.1155	0.56040	0.44435	0.17148
Treatment			0.0109			0.3774			
Palliative 1st Line	54 (35.3)	105 (42)		54 (35.3)	66 (43.1)		0.13804	0.18218	0.16145
Palliative 2nd Line	11 (7.2)	22 (8.8)		11 (7.2)	13 (8.5)		0.05941	0.01727	0.04822
Hematological cancer	49 (32)	44 (17.6)		49 (32)	37 (24.2)		−0.47211	−0.44009	−0.32843
Neoadj./Definit./Adjuv.	39 (25.5)	79 (31.6)		39 (25.5)	37 (24.2)		0.13559	0.06410	−0.02901
Primary Site			<.0001			0.0456			
GI	12 (7.8)	78 (31.2)		12 (7.8)	27 (17.7)		0.61665	0.44701	0.25883
Head and Neck	7 (4.6)	17 (6.8)		7 (4.6)	10 (6.5)		0.09617	0.13325	0.08476
Lung	15 (9.8)	21 (8.4)		15 (9.8)	15 (9.8)		−0.04882	−0.01198	0.00000
Others	5 (3.3)	14 (5.6)		5 (3.3)	10 (6.5)		0.11347	0.14784	0.15901
Breast	40 (26.1)	24 (9.6)		40 (26.1)	21 (13.7)		−0.44225	−0.44601	−0.33197
Gynecological	12 (7.8)	33 (13.2)		12 (7.8)	19 (12.4)		0.17526	0.21499	0.14968
Prostate	7 (4.6)	8 (3.2)		7 (4.6)	8 (5.2)		−0.07119	−0.05029	0.03383
Cent. Nervous Syst.	2 (1.3)	4 (1.6)		2 (1.3)	2 (1.3)		0.02447	0.00369	0.00000
Hematologic	49 (32)	44 (17.6)		49 (32)	37 (24.2)		−0.33875	−0.28663	−0.18417
Sarcomas	4 (2.6)	7 (2.8)		4 (2.6)	4 (2.6)		0.01144	0.03320	0.00000
Logit Prop. Score							1.16342	0.98048	0.53824
Outcome			0.0380			0.0709			
Hospital Discharge	60 (39.2)	73 (29.2)		60 (39.2)	45 (29.4)				
Death	93 (60.8)	177 (70.8)		93 (60.8)	108 (70.6)				

Abbreviations: Adjuv.: adjuvant; Cent.: Central; Definit.: Definitive; GI: Gastrointestinal; Neoadj.: Neoadjuvant; Syst.: System.

Table S6. Baseline variables before and after active chemotherapy (**alkylating**) use and non−active chemotherapy groups propensity score matching.

Characteristic	Non−Matched			Pair Matched			Standardized mean difference		

	Active Chemo. *n* = 115	Non−active Chemo. n = 250	*p*	Active Chemo. *n* = 108	Non−active Chemo. n = 108	*p*	All	Region	Matched
Age			<0.0001			0.2088			
<50 years	46 (40)	46 (18.4)		41 (38)	28 (25.9)		−0.48905	−0.36026	−0.27253
50 to 64 years	40 (34.8)	102 (40.8)		38 (35.2)	41 (38)		0.12434	0.12069	0.05740
65 to 79 years	25 (21.7)	93 (37.2)		25 (23.2)	31 (28.7)		0.34411	0.21527	0.12365
≥80 years	4 (3.5)	9 (3.6)		4 (3.7)	8 (7.4)		0.00659	0.02159	0.20045
Sex			0.2358			0.7818	0.13417	0.07604	0.03730
Male	48 (41.7)	121 (48.4)		43 (39.8)	45 (41.7)				
Female	67 (58.3)	129 (51.6)		65 (60.2)	63 (58.3)				
Comorbidities									
Heart Disease	17 (14.8)	60 (24)	0.0450	17 (15.7)	26 (24.1)	0.1251	0.23474	0.22601	0.21222
Diabetes	14 (12.2)	38 (15.2)	0.4423	14 (13)	15 (13.9)	0.8418	0.08813	0.04066	0.02697
Neurologic disease	2 (1.7)	8 (3.2)	0.4270	2 (1.9)	2 (1.9)	1.0000	0.09424	0.07902	0.00000
Chronic lung disease	4 (3.5)	12 (4.8)	0.5667	2 (1.9)	5 (4.6)	0.2490	0.06639	0.16457	0.13953
Nephropathy	9 (7.8)	16 (6.4)	0.6163	8 (7.4)	9 (8.3)	0.8005	−0.05550	0.01109	0.03604
Critical Presentation	29 (25.2)	61 (24.4)	0.8663	28 (25.9)	27 (25)	0.8759	−0.01893	0.00528	−0.02144
Southeast	76 (66.1)	138 (55.2)	0.0498	73 (67.6)	67 (62)	0.3926	−0.22424	−0.19864	−0.11443
Clinical Stage			<0.0001						
I/II	20 (17.4)	42 (16.8)		19 (17.6)	16 (14.8)		−0.03887	−0.05499	−0.03762
III/IV	49 (42.6)	172 (68.8)		49 (45.4)	58 (53.7)		0.07249	0.10761	−0.04385
Unknown	14 (12.2)	7 (2.8)		8 (7.4)	5 (4.6)		−0.36196	−0.14742	−0.10726
Other	32 (27.8)	29 (11.6)		32 (29.6)	29 (26.9)		−0.41662	−0.37892	−0.07132
Mult. Drug Regime	82 (71.3)	132 (52.8)	0.0009	75 (69.4)	60 (55.6)	0.0350	0.38846	0.34898	0.29157
Treatment			<0.0001			0.0288			
Palliative 1st Line	19 (16.5)	105 (42)		19 (17.6)	33 (30.6)		0.58335	0.39562	0.29680
Palliative 2nd Line	2 (1.7)	22 (8.8)		2 (1.9)	7 (6.5)		0.32005	0.21824	0.20985
Hematological cancer	66 (57.4)	44 (17.6)		59 (54.6)	44 (40.7)		−0.69382	−0.52491	−0.27739
Neoadj./Definit./Adjuv.	28 (24.4)	79 (31.6)		28 (25.9)	24 (22.2)		0.16209	0.22287	−0.08278
Primary Site			<0.0001			0.0490			
GI	1 (0.9)	78 (31.2)		1 (0.9)	1 (0.9)		0.90776	0.66296	0.00000
Head and Neck	0 (0)	17 (6.8)		0 (0)	0 (0)		.	.	.
Lung	1 (0.9)	21 (8.4)		1 (0.9)	3 (2.8)		0.36407	0.47589	0.08953
Others	5 (4.4)	14 (5.6)		5 (4.6)	14 (13)		0.05762	0.11733	0.38347
Breast	30 (26.1)	24 (9.6)		30 (27.8)	24 (22.2)		−0.44095	−0.41375	−0.14858
Gynecological	1 (0.9)	33 (13.2)		1 (0.9)	6 (5.6)		0.49682	0.54124	0.18654
Prostate	1 (0.9)	8 (3.2)		1 (0.9)	5 (4.6)		0.16563	0.22577	0.26322
Cent. Nervous Syst.	6 (5.2)	4 (1.6)		6 (5.6)	4 (3.7)		−0.20036	−0.19409	−0.10257
Hematologic	66 (57.4)	44 (17.6)		59 (54.6)	44 (40.7)		−0.90160	−0.72655	−0.31470

Sarcomas	4 (3.5)	7 (2.8)		4 (3.7)	7 (6.5)		−0.03890	−0.00654	0.15933
Logit Prop. Score							0.99125	0.49339	0.14874
Outcome			0.0003			0.0007			
Hospital Discharge	56 (48.7)	73 (29.2)		52 (48.2)	28 (25.9)				
Death	59 (51.3)	177 (70.8)		56 (51.9)	80 (74.1)				

Abbreviations: Adjuv.: adjuvant; Cent.: Central; Definit.: Definitive; GI: Gastrointestinal; Neoadj.: Neoadjuvant; Syst.: System.

Table S7. Baseline variables before and after active chemotherapy (**cisplatin**) use and non−active chemotherapy groups propensity score matching.

Characteristic	Non−Matched			Pair Matched			Standardized mean difference		
	**Active Chemo. *n* = 186**	**Non−active Chemo. n = 250**	***p***	**Active Chemo. *n* = 186**	**Non−active Chemo. n = 186**	***n***	**All**	**Region**	**Matched**
Age			0.4874			0.7450			
< 50 years	37 (19.9)	46 (18.4)		37 (19.9)	36 (19.4)		−0.03794	−0.01636	−0.01367
50 to 64 years	86 (46.2)	102 (40.8)		86 (46.2)	80 (43)		−0.10982	−0.10891	−0.06516
65 to 79 years	56 (30.1)	93 (37.2)		56 (30.1)	65 (35)		0.15052	0.14817	0.10269
≥ 80 years	7 (3.8)	9 (3.6)		7 (3.8)	5 (2.7)		−0.00868	−0.05026	−0.05710
Sex			0.7410			10.000	−0.03201	−0.04226	0.00000
Male	93 (50)	121 (48.4)		93 (50)	93 (50)				
Female	93 (50)	129 (51.6)		93 (50)	93 (50)				
Comorbidities									
Heart Disease	38 (20.4)	60 (24)	0.3771	38 (20.4)	38 (20.4)	1	0.08596	0.01677	0.00000
Diabetes	25 (13.4)	38 (15.2)	0.6054	25 (13.4)	27 (14.5)	0.7649	0.05024	0.01838	0.03071
Neurologic disease	3 (1.6)	8 (3.2)	0.2959	3 (1.6)	4 (2.2)	0.7028	0.10370	0.07867	0.03513
Chronic lung disease	11 (5.9)	12 (4.8)	0.6068	11 (5.9)	10 (5.4)	0.8223	−0.04949	−0.03330	−0.02388
Nephropathy	8 (4.3)	16 (6.4)	0.3419	8 (4.3)	9 (4.8)	0.8039	0.09337	0.05929	0.02392
Critical Presentation	41 (22)	61 (24.4)	0.5653	41 (22)	45 (24.2)	0.6228	0.05584	0.04503	0.05095
Southeast	112 (60.2)	138 (55.2)	0.2950	112 (60.2)	102 (54.8)	0.2942	−0.10165	−0.11664	−0.10897
Clinical Stage			0.0138			0.4355			
I/II	32 (17.2)	42 (16.8)		32 (17.2)	42 (22.6)		−0.01869	0.02445	0.06676
III/IV	140 (75.3)	172 (68.8)		140 (75.3)	135 (72.6)		0.03941	0.07435	0.07559
Unknown	8 (4.3)	7 (2.8)		8 (4.3)	6 (3.2)		−0.08118	−0.08027	−0.05815
Other	6 (3.2)	29 (11.6)		6 (3.2)	3 (1.6)		0.32381	0.07496	−0.06237
Mult. Drug Regime	133 (71.5)	132 (52.8)	<.0001	133 (71.5)	112 (60.2)	0.0217	0.39305	0.25952	0.23724
Treatment			<.0001			0.0261			
Palliative 1st Line	119 (64)	105 (42)		119 (64)	90 (48.4)		−0.45144	−0.41730	−0.32025
Palliative 2nd Line	5 (2.7)	22 (8.8)		5 (2.7)	8 (4.3)		0.26496	0.18876	0.06992
Hematological cancer	14 (7.5)	44 (17.6)		14 (7.5)	21 (11.3)		0.11608	0.12563	0.05946
Neoadj./Definit./Adjuv.	48 (25.8)	79 (31.6)		48 (25.8)	67 (36)		0.12833	0.20833	0.22627
Primary Site			<.0001			0.4996			
GI	84 (45.2)	78 (31.2)		84 (45.2)	74 (39.8)		−0.29038	−0.18742	−0.11182
Head and Neck	14 (7.5)	17 (6.8)		14 (7.5)	14 (7.5)		−0.02819	−0.00059	0.00000

Lung	25 (13.4)	21 (8.4)		25 (13.4)	20 (10.8)		−0.16215	−0.11521	−0.08647
Others	10 (5.4)	14 (5.6)		10 (5.4)	12 (6.5)		0.00982	0.05253	0.04721
Breast	6 (3.2)	24 (9.6)		6 (3.2)	13 (7)		0.26242	0.23443	0.15494
Gynecological	32 (17.2)	33 (13.2)		32 (17.2)	32 (17.2)		−0.11170	−0.04774	0.00000
Prostate	1 (0.5)	8 (3.2)		1 (0.5)	0 (0)		0.19756	0.06462	−0.03989
Cent. Nervous Syst.	0 (0)	4 (1.6)		0 (0)	0 (0)		.	.	.
Hematologic	14 (7.5)	44 (17.6)		14 (7.5)	21 (11.3)		0.30749	0.20018	0.11488
Sarcomas	0 (0)	7 (2.8)		0 (0)	0 (0)		.	.	.
Logit Prop. Score							0.52847	0.17099	0.11163
Outcome			0.3559			0.5784			
Hospital Discharge	62 (33.3)	73 (29.2)		62 (33.3)	57 (30.7)				
Death	124 (66.7)	177 (70.8)		124 (66.7)	129 (69.4)				

Abbreviations: Adjuv.: adjuvant; Cent.: Central; Definit.: Definitive; GI: Gastrointestinal; Neoadj.: Neoadjuvant; Syst.: System.

Table S8. Baseline variables before and after active chemotherapy (**topoisomerase I inhibitor**) use and non−active chemotherapy groups propensity score matching.

	**Non−Matched**			**Pair Matched**			**Standardized mean difference**		
**Characteristic**	**Active Chemo. Group *n* = 115**	**Non−active Chemo. n = 250**	***p***	**Active Chemo. Group n = 34**	**Non−active Chemo. n = 34**	***p***	**All**	**Region**	**Matched**
Age			0.3997			0.8967			
< 50 years	11 (30.6)	46 (18.4)		10 (29.4)	8 (23.5)		−0.28558	−0.20580	−0.13820
50 to 64 years	13 (36.1)	102 (40.8)		12 (35.3)	12 (35.3)		0.09649	−0.03290	0.00000
65 to 79 years	11 (30.6)	93 (37.2)		11 (32.4)	12 (35.3)		0.14073	0.16657	0.06230
≥ 80 years	1 (2.8)	9 (3.6)		1 (2.9)	2 (5.9)		0.04681	0.14196	0.16744
Sex			0.8575			0.6252	−0.03201	−0.11512	−0.11768
Male	18 (50)	121 (48.4)		16 (47.1)	14 (41.2)				
Female	18 (50)	129 (51.6)		18 (52.9)	20 (58.8)				
Comorbidities									
Heart Disease	4 (11.1)	60 (24)	0.0828	4 (11.8)	6 (17.7)	0.4935	0.34376	0.15007	0.15689
Diabetes	8 (22.2)	38 (15.2)	0.2836	6 (17.7)	4 (11.8)	0.4935	−0.18079	0.10535	−0.15144
Neurologic disease	0 (0)	8 (3.2)	0.2763	0 (0)	0 (0)	1.0000	.	.	.
Chronic lung disease	1 (2.8)	12 (4.8)	0.5860	1 (2.9)	1 (2.9)	1.0000	0.10606	0.01677	0.00000
Nephropathy	1 (2.8)	16 (6.4)	0.3901	1 (2.9)	0 (0)	0.3137	0.17376	0.01534	−0.14109
Critical Presentation	7 (19.4)	61 (24.4)	0.5138	7 (20.6)	4 (11.8)	0.3232	0.12000	0.00155	−0.21366
Southeast	24 (66.7)	138 (55.2)	0.1943	23 (67.7)	23 (67.7)	1.0000	−0.23666	−0.20718	0.00000
Clinical Stage			0.7303			0.3062			
I/II	4 (11.1)	42 (16.8)		3 (8.8)	0 (0)		−0.09055	0.02481	−0.22829
III/IV	25 (69.4)	172 (68.8)		25 (73.5)	28 (82.4)		0.45419	−0.00353	0.00000
Unknown	1 (2.8)	7 (2.8)		1 (2.9)	2 (5.9)		0.00135	0.08543	0.17863
Other	6 (16.7)	29 (11.6)		5 (14.7)	4 (11.8)		−0.14583	0.13986	−0.08465
Mult. Drug Regime	22 (61.1)	132 (52.8)	0.3497	20 (58.8)	19 (55.9)	0.8063	0.16845	−0.08553	0.05961
Treatment			<.0001			0.5517			

Palliative 1st Line	16 (44.4)	105 (42)		16 (47.1)	20 (58.8)		−0.04936	0.12523	0.23756
Palliative 2nd Line	11 (30.6)	22 (8.8)		10 (29.4)	9 (26.5)		−0.56893	−0.42805	−0.07692
Hematological cancer	9 (25)	44 (17.6)		8 (23.5)	5 (14.7)		−0.28218	−0.00733	−0.33722
Neoadj./Definit./Adjuv.	0 (0)	79 (31.6)		0 (0)	0 (0)		.	.	.
Primary Site			0.0070			0.5919			
GI	22 (61.1)	78 (31.2)		21 (61.8)	22 (64.7)		−0.62897	−0.29310	0.06185
Head and Neck	0 (0)	17 (6.8)		0 (0)	0 (0)		.	.	.
Lung	0 (0)	21 (8.4)		0 (0)	0 (0)		.	.	.
Others	0 (0)	14 (5.6)		0 (0)	0 (0)		.	.	.
Breast	0 (0)	24 (9.6)		0 (0)	0 (0)		.	.	.
Gynecological	5 (13.9)	33 (13.2)		5 (14.7)	7 (20.6)		−0.02013	0.11025	0.17191
Prostate	0 (0)	8 (3.2)		0 (0)	0 (0)		.	.	.
Cent. Nervous Syst.	0 (0)	4 (1.6)		0 (0)	0 (0)		.	.	.
Hematologic	9 (25)	44 (17.6)		8 (23.5)	5 (14.7)		−0.18148	0.24932	−0.21639
Sarcomas	0 (0)	7 (2.8)		0 (0)	0 (0)		.	.	.
Logit Prop. Score							1.50208	0.05071	0.01030
Outcome			0.1297			0.0381			
Hospital Discharge	15 (41.7)	73 (29.2)		15 (44.1)	7 (20.6)				
Death	21 (58.3)	177 (70.8)		19 (55.9)	27 (79.4)				

Abbreviations: Adjuv.: adjuvant; Cent.: Central; Definit.: Definitive; GI: Gastrointestinal; Neoadj.: Neoadjuvant; Syst.: System.

Table S9. Baseline variables before and after active chemotherapy (topoisomerase II inhibitor) use and non−active chemotherapy groups propensity score matching.

Characteristic	Non−Matched			Pair Matched			Standardized Mean Difference		
	Active Chemo. Group *n* = 94	Non−Active Chemo. *n* = 250	*p*	Active Chemo. Group *n* = 80	Non−Active Chemo. *n*=80	*p*	All	Region	Matched
Age			<0.0001			0.1384			
<50 years	48 (51.1)	46 (18.4)		35 (43.8)	21 (26.3)		−0.73036	−0.41925	−0.39130
50 to 64 years	27 (28.7)	102 (40.8)		26 (32.5)	35 (43.8)		0.25566	0.17936	0.23816
65 to 79 years	16 (17)	93 (37.2)		16 (20)	21 (26.3)		0.46610	0.22778	0.14437
≥80 years	3 (3.2)	9 (3.6)		3 (3.8)	3 (3.8)		0.02256	0.02301	0.00000
Sex			0.0422			0.6230	0.24948	0.10766	0.07650
Male	34 (36.2)	121 (48.4)		28 (35)	31 (38.8)				
Female	60 (63.8)	129 (51.6)		52 (65)	49 (61.3)				
Comorbidities									
Heart Disease	14 (14.9)	60 (24)	0.0670	13 (16.3)	18 (22.5)	0.3173	0.23162	0.27554	0.15897
Diabetes	10 (10.6)	38 (15.2)	0.2765	9 (11.3)	11 (13.8)	0.6326	0.13632	0.12036	0.07471
Neurologic disease	1 (1.1)	8 (3.2)	0.2687	1 (1.3)	1 (1.3)	1.0000	0.14829	0.05785	0.00000
Chronic lung disease	3 (3.2)	12 (4.8)	0.5150	2 (2.5)	4 (5)	0.4053	0.08220	0.15614	0.12775
Nephropathy	3 (3.2)	16 (6.4)	0.2457	2 (2.5)	6 (7.5)	0.1468	0.15058	0.14340	0.23466
Critical Presentation	23 (24.5)	61 (24.4)	0.9895	20 (25)	23 (28.8)	0.5926	−0.00158	0.12929	0.08727

Southeast	64 (68.1)	138 (55.2)	0.0305	50 (62.5)	48 (60)	0.7455	−0.26734	−0.11527	−0.05187
Clinical Stage			<0.0001			0.4955			
I/II	16 (17)	42 (16.8)		15 (18.8)	12 (15)		0.02947	0.03006	0.00000
III/IV	41 (43.6)	172 (68.8)		41 (51.3)	43 (53.8)		0.05797	0.06875	−0.05893
Unknown	11 (11.7)	7 (2.8)		8 (10)	4 (5)		−0.34844	−0.20114	−0.19571
Other	26 (27.7)	29 (11.6)		16 (20)	21 (26.3)		−0.41285	−0.06784	0.16067
Mult. Drug Regime	86 (91.5)	132 (52.8)	<0.0001	72 (90)	62 (77.5)	0.0321	0.95671	0.45678	0.30910
Treatment			<0.0001			0.1046			
Palliative 1st Line	10 (10.6)	105 (42)		10 (12.5)	22 (27.5)		0.76213	0.50628	0.36452
Palliative 2nd Line	1 (1.1)	22 (8.8)		1 (1.3)	1 (1.3)		0.36311	0.07171	0.00000
Hematological cancer	56 (59.6)	44 (17.6)		43 (53.8)	39 (48.8)		−1.09115	−0.75341	−0.42761
Neoadj./Definit./Adjuv.	27 (28.7)	79 (31.6)		26 (32.5)	18 (22.5)		0.06271	0.01817	−0.21799
Primary Site			<0.0001			0.3504			
GI	1 (1.1)	78 (31.2)		1 (1.3)	1 (1.3)		0.89813	0.50084	0.00000
Head and Neck	0 (0)	17 (6.8)		0 (0)	0 (0)		.	.	.
Lung	2 (2.1)	21 (8.4)		2 (2.5)	5 (6.3)		0.28369	0.32665	0.16961
Others	2 (2.1)	14 (5.6)		2 (2.5)	6 (7.5)		0.18090	0.26772	0.26049
Breast	28 (29.8)	24 (9.6)		27 (33.8)	19 (23.8)		−0.52481	−0.44411	−0.25997
Gynecological	1 (1.1)	33 (13.2)		1 (1.3)	3 (3.8)		0.48525	0.44982	0.09996
Prostate	0 (0)	8 (3.2)		0 (0)	0 (0)		.	.	.
Cent. Nervous Syst.	0 (0)	4 (1.6)		0 (0)	0 (0)		.	.	.
Hematologic	56 (59.6)	44 (17.6)		43 (53.8)	39 (48.8)		−0.95562	−0.52806	−0.11383
Sarcomas	4 (4.3)	7 (2.8)		4 (5)	7 (8.8)		−0.07895	−0.00753	0.20343
Logit Prop. Score							1.25126	0.42208	0.16195
Outcome			<0.0001			<0.0001			
Hospital Discharge	52 (55.3)	73 (29.2)		46 (57.5)	20 (25)				
Death	42 (44.7)	177 (70.8)		34 (42.5)	60 (75)				

Abbreviations: Adjuv.: adjuvant; Cent.: Central; Definit.: Definitive; GI: Gastrointestinal; Neoadj.: Neoadjuvant; Syst.: System.

© 2020 by the authors. Licensee MDPI, Basel, Switzerland. This article is an open access article distributed under the terms and conditions of the Creative Commons Attribution (CC BY) license (http://creativecommons.org/licenses/by/4.0/).
